# The Implication of a Polymorphism in the Methylenetetrahydrofolate Reductase Gene in Homocysteine Metabolism and Related Civilisation Diseases

**DOI:** 10.3390/ijms25010193

**Published:** 2023-12-22

**Authors:** Emilia Zarembska, Klaudia Ślusarczyk, Małgorzata Wrzosek

**Affiliations:** 1 Student Scientific Association “Farmakon”, Department of Biochemistry and Pharmacogenomics, Medical University of Warsaw, 1 Banacha St., 02-097 Warsaw, Poland; 2 Department of Medical Genetics, Institute of Mother and Child, 17a Kasprzaka St., 01-211 Warsaw, Poland; 3 Department of Biochemistry and Pharmacogenomics, Medical University of Warsaw, 1 Banacha St., 02-097 Warsaw, Poland; 4 Centre for Preclinical Research, Medical University of Warsaw, 1B Banacha St., 02-097 Warsaw, Poland

**Keywords:** folate, gene variants, MTHFR

## Abstract

Methylenetetrahydrofolate reductase (MTHFR) is a key regulatory enzyme in the one-carbon cycle. This enzyme is essential for the metabolism of methionine, folate, and RNA, as well as for the production of proteins, DNA, and RNA. MTHFR catalyses the irreversible conversion of 5,10-methylenetetrahydrofolate to its active form, 5-methyltetrahydrofolate, a co-substrate for homocysteine remethylation to methionine. Numerous variants of the *MTHFR* gene have been recognised, among which the C677T variant is the most extensively studied. The C677T polymorphism, which results in the conversion of valine to alanine at codon 222, is associated with reduced activity and an increased thermolability of the enzyme. Impaired MTHFR efficiency is associated with increased levels of homocysteine, which can contribute to increased production of reactive oxygen species and the development of oxidative stress. Homocysteine is acknowledged as an independent risk factor for cardiovascular disease, while chronic inflammation serves as the common underlying factor among these issues. Many studies have been conducted to determine whether there is an association between the C677T polymorphism and an increased risk of cardiovascular disease, hypertension, diabetes, and overweight/obesity. There is substantial evidence supporting this association, although several studies have concluded that the polymorphism cannot be reliably used for prediction. This review examines the latest research on *MTHFR* polymorphisms and their correlation with cardiovascular disease, obesity, and epigenetic regulation.

## 1. Methylenetetrahydrofolate Reductase (MTHFR)

Methylenetetrahydrofolate reductase (MTHFR) is an enzyme encoded by the *MTHFR* gene composed of 12 exons and located on chromosome 1p36.22. Its total length is 20,374 bp [1]. Characteristic elements, such as SP1, AP1, AP2, CAAT, or GC, are involved in the regulation of the expression of this gene, but there are no TATA-box elements. The structure of this promoter region is shared by other genes involved in homocysteine (Hcy) metabolism, including cystathionine β-synthase (*CBS*), methionine synthase (*MS*), and methionine synthase reductase coding genes [2].

During *MTHFR* transcription, alternative splicing occurs, resulting in three different mRNA molecules of 7074, 7018, and 7071 bp, which encode polypeptides of 697, 656, and 696 amino acids, respectively [1]. On this basis, three variants of the human *MTHFR* transcript have been distinguished: *MTHFR* 1, 2, and 3, which differ at the end of 5′ [3]. As mentioned above, the presence of the transcript’s different sizes was due to alternative transcription start sites and the use of polyadenylation signal sequences [4]. Western blot analysis discovered a major polypeptide of approximately 77 kDa in human tissues, while a protein isoform of approximately 70 kDa was detected only in human liver tissue [5,6].

**Figure 1:**
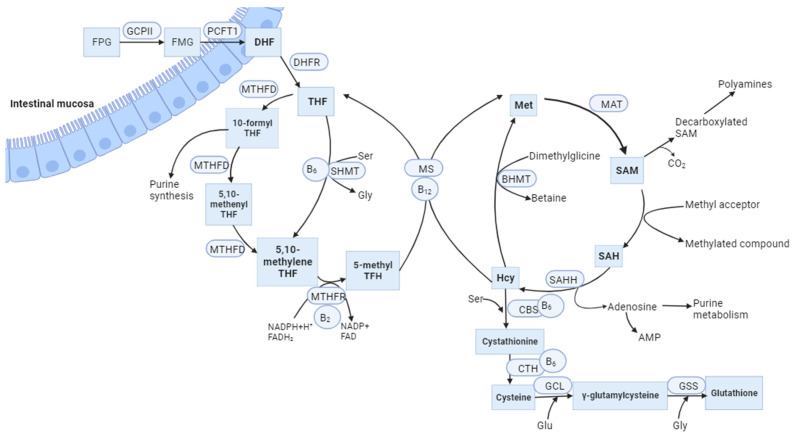
The balance between the folate cycle and the methionine cycle is affected by vitamin B_12_. FPG, Folate polyglutamates; FMG, Folate monoglutamates; GCPII, glutamate carboxypeptidase II; PCFT1, proton-coupled folate transporter; DHF, dihydrofolate; DHRF, dihydrofolate reductase; THF, tetrahydrofolate; MTHFD, methylenetetrahydrofolate dehydrogenase; SHMT, serinehydroxymethyl transferase; Ser, Serine; Gly, Glycine; MTHFR, methylenetetrahydrofolate reductase; NADPH, nicotinamide adenine dinucleotide phosphate; FADH_2_, dihydroflavine-adeninedinucleotide; MS, methionine synthase; Met, Methionine; MAT, methionine adenosyltransferase; SAM, S-adenosylmethionine; SAH, S-adenosylhomocysteine; SAHH, S-adenosylhomocysteine hydrolase; BHMT, Betaine-homocysteine methyltransferase; CBS, Cystathionine-β-synthase; CTH, Cystathionine gamma lyase; GCL, glutamate cysteine ligase; GSS, Glutathione synthetase; Glu, Glutamic acid; B_2_, vitamin B_2_ (riboflavin); B_6_, vitamin B_6_ (pyridoxine); B_12_, vitamin B_12_ (cobalamin).

MTHFR is an FAD-dependent enzyme that plays a significant role in the metabolism of folate and Hcy, both of which are based on folic acid and other vitamins in the B group. This enzyme catalyses the NADPH-linked reduction of 5,10-methylenetetrahydrofolate (5,10-methylene-THF) to 5-methyltetrahydrofolate (5-methyl-THF). The last molecule is a methyl group donor for the conversion of Hcy to methionine (Met) in the reaction catalysed by methionine synthase (MS) (Figure 1). Vitamin B_12_ acts as a cofactor during this process. Met is then converted to S-adenosylmethionine (SAM), which is a crucial methyl group donor for various reactions in the body, including the methylation of DNA [7], RNA, histones, phospholipids, choline, sphingomyelin, acetylcholine, and other neurotransmitters [8]. Protein carboxymethylation may be involved in the repair of ageing proteins; also, heat shock proteins are methylated in response to stress [9]. In addition, Hcy can be converted to cysteine through a trans-sulphuration process involving the enzyme CBS and vitamin B_6_. Under Met deficiency conditions, CBS is not activated and MTHFR is not inhibited by SAM. As a result, Hcy is converted back into Met, while cysteine contributes to glutathione synthesis or is degraded to taurine (Figure 1) [10].

## 2. *MTHFR* Polymorphism

The first documentation of MTHFR involvement in disease came from the research conducted by Mudd et al. in 1972 [11]. They identified patients with homocystinuria, which was attributed to a significant deficiency in MTHFR obtained from fibroblasts. Kang et al. in 1988 described decreased activity and increased thermolability of the MTHFR enzyme in lymphocyte extracts obtained from patients with ischemic heart disease (IHD) [12]. Some of them had a decrease in enzyme activity by up to 75% and an increase in total Hcy levels, but this was not the case in patients with high levels of folate and vitamin B_12_ [13]. The variations observed between individuals suggest the existence of genetic diversity within *MTHFR*. Currently, several dozen polymorphisms have been identified, with the most studied being the C677T (rs1801133) and the A1298C polymorphism (rs180113) [14,15]. The first one involves a substitution of cytosine to thymine at position 677 within exon 4, leading to a substitution of alanine to valine at position 222 within the catalytic domain of the MTHFR. This site is crucial in terms of the binding of flavin adenine dinucleotides (FAD) and enzyme stability. The 677T allele encodes a thermolabile enzyme with reduced activity and less affinity for its cofactor, FAD. Each copy of the 677T allele results in 35% reduced enzyme activity. The 677TT homozygotes are believed to have reduced levels of active folate (5-methyl-THF) and increased plasma levels of Hcy because it cannot be remethylated to Met [6].

The prevalence of the C677T polymorphism varies by ethnic group and geographic location and has a relatively high frequency worldwide. A meta-analysis of population-based studies revealed that the worldwide prevalence of the T allele was estimated to be 24.0%, while the global occurrence of the TT genotype was 7.7%. However, upon closer examination of different subgroups, it became evident that the prevalence of the T allele exhibited significant variation: 10.3% in Africans, 31.2% in North Americans, 27.8% in South Americans, 19.7% in Asians, 20.5% in Australians, and 34.1% in Europeans. The prevalence of both the T allele and the TT genotype was lowest among Africans and highest among Europeans. The occurrence of the T allele differs significantly between the Asian population. Research carried out within Asian populations showed that this particular gene polymorphism showed a notably higher prevalence in East Asian countries (44.7% in China, 40.3% in the Republic of Korea, and 39.9% in Japan). On the contrary, South Asian countries had a lower prevalence (11.4% in India, 16% in Pakistan, and 4.5% in Sri Lanka) [16].

Another common *MTHFR* polymorphism is 1298A>C. However, the presence of this variant does not lead to elevated levels of Hcy in heterozygous or homozygous individuals. Instead, the combined heterozygosity of 1298A>C and 677C>T produces similar results to having a TT genotype [17].

## 3. Regulation of MTHFR Activity

**Figure 2:**
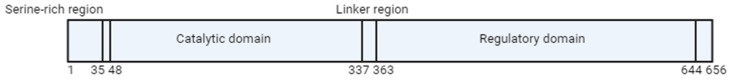
Schematic representation of MTHFR protein. The numbers given represent amino acids in human MTHFR.

Folate metabolism and the methionine cycle share a common step involving the MTHFR reaction, so regulation of reductase activity is crucial for maintaining reference Met and SAM concentrations in cells. MTHFR activity is inhibited by dihydrofolate (DHF) and its polyglutamate analogues [18]. The human MTHFR protein comprises multiple domains, including an N-terminal catalytic domain, which houses a conserved serine-rich region, and a C-terminal regulatory domain (Figure 2). The catalytic domain is connected to the regulatory domain by a linker sequence (“linker region”). The regulatory domain plays a role in binding to SAM, which subsequently functions as an allosteric inhibitor of reductase activity [19]. The effect of the inhibition reaction is very slow and can be reversed by the attachment of SAH, the demethylated form of SAM [20].

The enzyme activity is also affected by multiple phosphorylation of the serine-rich region [21]. One of the phosphorylation sites is Thr 34. The substitution of threonine to alanine at position 34 completely blocks phosphorylation, suggesting that Thr 34 serves as the initiation site for this process. Yamada et al. [20] expressed the mutant Thr34Ala in baculovirus-infected insect cells infected with baculoviruses and compared its enzymatic properties with the wild-type enzyme. MTHFR was treated with alkaline phosphatase, which removes seven phosphoryl groups from the enzyme. The wild-type exposed to alkaline phosphatase and the mutant enzyme had higher activity compared to the wild-type enzyme not treated with phosphatase, and they were also less sensitive to inhibition by SAM [22]. Phosphorylation probably protects SAM from spontaneous degradation to SAH and may induce a conformational change in the MTHFR enzyme. Furthermore, SAM may affect the stability of the linkage to FAD, an essential cofactor of MTHFR. The presence of mutations or polymorphisms in the *MTHFR* gene that affect the function of FAD may have similar effects [21].

The molecular basis of the enzymatic regulation remained unknown until 2018. The article by Froese et al. identified the aforementioned “linker region”, which links two processes: SAM binding to the regulatory domain and inhibition in the catalytic domain (Figure 2). Moreover, these processes are individually mediated by regions more than 300 amino acids apart [21].

An indicator of the ability of cells to methylate DNA or form compounds requiring methyl groups is the SAM/SAH ratio. Under conditions of low SAM/SAH ratio (methyl donor deficiency), MTHFR is activated, leading to an increase in the concentration of active folate (5-methyl-THF). As a result, the concentration of SAM in the cell increases. A high SAM/SAH ratio leads to efficient and effective methylation, resulting in SAM-mediated allosteric inhibition of the enzyme, which reduces the concentration of 5-methyl-THF and decreases the activity of the methionine cycle, and thus also the production of SAM [21].

Observations of MTHFR regulation by phosphorylation were validated in a study by Zheng et al. [23].They investigated the activity of the DYRK1A/2 and GSK3A/B kinases responsible for multisite phosphorylation of MTHFR and its physiological significance in cells. To confirm that the phosphorylated MTHFR is less active than the non-phosphorylated form, under physiological SAM concentration conditions (1–3 μM), mutant MTHFR knock-in lines were created using the CRISPR method (Clustered Regularly Interspaced Short Palindromic Repeats). The enzyme with a mutation (completely devoid of phosphorylation) was compared to the original parental cell lines. The parental cell lines showed an increase in 5-methyl-THF production in response to Hcy treatment, while the knockin cell lines had high basal levels of 5-methyl-THF and did not respond to Hcy treatment. The results suggest that multisite phosphorylation of the MTHFR enzyme is associated with SAM attachment to inhibit MTHFR activity in cells [23]. 

In summary, the physiological function of multisite phosphorylation is considered to be as follows: under conditions of high Met concentration, it provides maximum inhibition of MTHFR by SAM, so that one-carbon units are “spared” for key processes, such as purine and deoxythymidine monophosphate (dTMP) synthesis. On the contrary, under low Met concentration conditions, MTHFR is dephosphorylated and becomes more active, leading to a diversion of more one-carbon units into the Met and SAM synthesis pathway. Dephosphorylation and activation of MTHFR may also serve as a cellular response to hyperhomocysteinemia (HHcy), ensuring sufficient concentrations of active folate for the remethylation of Hcy and the elimination of toxic effects caused by this amino acid [23].

## 4. Folate, Vitamin B_12_, and Homocysteine Metabolism 

Vitamin B_9_ is an essential exogenous microelement that naturally occurs in the form of folates. Folates are a family of chemically similar compounds that are key cofactors involved in the metabolism of one-carbon molecules. Folates include folic acid and its derivatives: 5-methyltetrahydrofolate (5-methyl-THF), 5-formyltetrahydrofolate (5-formyl-THF or folinic acid), 10-formyltetrahydrofolate (10-formyl-THF), 5,10-methylenetetrahydrofolate (5,10-methylene-THF), and unsubstituted THF (tetrahydrofolate). The main biologically active metabolite of ingested folic acid is 5-methyl-THF (5-MTHF), known as levomepholic acid. This form is found in plasma at the highest concentration and accounts for >90% of total folate concentration. Most dietary folate is converted to 5-methyl-THF before entering the bloodstream [24,25]. The estimated body content of folate is approximately 10 mg to 30 mg, while the normal serum level of total folate is approximately 5 to 15 ng/mL [26]. Inadequate folate levels hinder DNA replication and cell division, detrimentally impacting rapidly dividing tissues, such as bone marrow, and leading to a decrease in blood cell production [27].

Folic acid is the synthetic, water-soluble form of vitamin B_9_, also known as pteroylmonoglutamic acid. Chemically, it consists of p-aminobenzoic acid, glutamic acid, and a pteridine base. Folic acid itself cannot function as a coenzyme and must be reduced to DHF and then to THF in a two-step enzymatic reaction. Unlike naturally occurring folate, not all folic acid supplied from fortified food is converted to 5-MTHF. The benefit of folic acid supplementation is well established in the preconception period for the prevention of neural tube defects. However, folic acid supplementation may lead to the accumulation of unmetabolized folic acid (UMFA) in systemic circulation with potential toxic effects [26]. Consuming this synthetic form in excess of 200 μg per meal is suggested to exceed the dihydrofolate reductase capacity (DHFR) to convert folic acid into THF [28]. In fact, the metabolic and biological outcomes of the persistence of UMFA in the bloodstream are currently undetermined. 

A defect in folate metabolism involving the presence of the variant C677T, which leads to reduced MTHFR activity, is correlated with lowered blood folate levels [29]. Individuals with severe MTHFR deficiency are characterised by highly impaired production of 5-MTHF. A study by Nishio et al. [30] in a Japanese population aged 20 to 73 years showed that folate concentrations were significantly lower in those with the TT genotype than in those with the CC genotype after the same folate supplementation. The researchers suggested that the folate requirement of TT homozygotes may be 1.4 times higher compared to those with 677CC/CT genotypes [30]. In addition, an association has been observed between elevated Hcy levels and reduced levels of folate and vitamin B_12_ among patients with severe coronary atherosclerosis [31]. The *MTHFR* C677T polymorphism has also been shown to alter the composition of folate derivatives in erythrocytes. Patients with the CC genotype had folate only in the form of 5-methyl-THF, while in those with the TT genotype, the concentration of folate in red blood cells also consisted of formylated polyglutamate THF. The proportion of formyl-THF in TT homozygotes ranged from 0 to 59% of the total folate in erythrocytes [32]. These results confirm that the thermolabile form of MTHFR catalyses the synthesis of 5-MTHF less efficiently compared to the normal form of the enzyme. Furthermore, additional polymorphisms that influence folate metabolism include: *MTRR* A66G, *MTHFR* A1298C, *MTR* A2756G, *CBS* 844ins68, and *GCPII* H475Y [33].

Folates enable the transfer of one-carbon groups (including methyl, methylene, or formyl) in reactions necessary for the synthesis of nucleic acid precursors: purines, pyrimidines, and dTMP. They participate in Met, serine, glycine, and histidine metabolism, and they are essential for the formation of methylating compounds responsible for proper metabolism and regulation of gene expression [34]. Moreover, folate compounds are crucial for all cells in the body, as they enable their normal growth, development, and reproduction.

Folate, which is present in foods as a polyglutamate conjugate, undergoes hydrolysis in the intestinal mucosa to its monoglutamate form, which serves as the transport form of folate (Figure 1). The active form, as mentioned earlier, is 5-MTHF, which functions intracellularly as a methyl group donor in the Hcy remethylation reaction. The conversion of THF (the reduced form of folic acid) to 5,10-methylene-THF by 10-formyl-THF and 5,10-methenyl-THF is catalysed by the trifunctional enzyme methylene-THF dehydrogenase (MTHFD), which exhibits the activities of formyl-THF synthase, methenyl-THF cyclohydrolase, and methenyl-THF dehydrogenase [35]. THF can be directly converted to 5,10-methylene-THF by the vitamin B_6_-dependent enzyme serine hydroxymethyltransferase (SHMT), resulting in the formation of glycine and 5,10-methylene-THF. The latter is then converted to 5-methyl-THF with the participation of MTHFR (Figure 1) [36,37]. This irreversible reaction catalysed by MTHFR is crucial in the folate metabolism cycle because it regulates the bioavailability of 5-methyl-THF, which is required for Met synthesis. Met, on the other hand, can be metabolised to SAM, which functions as the main methyl group donor, which is important in the methylation processes of DNA, histones, and other proteins [38]. 10-formyl-THF can act as a carbon donor for purine synthesis. 5,10-Methyl-THF donates a methylene group in the conversion reaction of dUMP to dTMP. In this reaction, DHF is formed, which is then reduced by DHFR to THF [39]. 

Vitamin B_12_ is a compound belonging to the cobalamin group, and it is one of the eight water-soluble B vitamins. It must be supplied through food because the body cannot synthesise it. Vitamin B_12_ absorption occurs in the terminal ileum with the help of an intrinsic factor. It is stored mainly in the liver, and its reserves last for 5-10 years. Reduced intake, malabsorption, or a rare, inborn defect in vitamin B_12_ metabolism are the main causes of deficiency. Vitamin B_12_ is responsible for proper erythropoiesis; it is also involved in DNA and RNA synthesis and carbohydrate and fat metabolism [40]. Some studies have found that vitamin B_12_ deficiency is associated with obesity. An example is a study by Baltaci et al. that examined the relationship between overweight, obesity, insulin resistance, metabolic syndrome (MetS), and vitamin B_12_ concentrations. Mean vitamin B_12_ concentrations among 976 individuals were significantly lower in overweight and obese patients compared to normal-weight patients. Furthermore, lower vitamin levels were shown in MetS patients and patients with insulin resistance, but these results were not statistically significant. In addition, a negative correlation was observed between vitamin B_12_ levels and BMI [41]. Similar results were obtained in a study that examined the relationship between vitamin B_12_ concentrations and BMI in 116 obese, middle-aged women. Women with obesity had lower vitamin B_12_ concentrations (244.1 ± 131.5 pg/mL) compared to control patients (336.2 ± 163.1 pg/mL). Therefore, an inverse correlation has also been observed between vitamin B_12_ levels and BMI [42]. Based on this, it can be assumed that overweight and obesity are risk factors that contribute to vitamin B_12_ deficiency. Therefore, it is recommended that overweight individuals increase their intake of this vitamin. Vitamin B_12_ acts as a cofactor for many enzymes, including MS, which catalyses the conversion of Hcy to Met. Therefore, its metabolism is closely related to folate metabolism. A deficiency in vitamin B_12_, just like a deficiency in folate, has been shown to cause higher levels of Hcy in the body. This is primarily because the conversion process from Hcy to Met is not carried out effectively [43].

Hcy (2-amino-4-mercaptobutyric acid) is an amino acid formed by the demethylation of Met, and it contains a thiol group in its structure. For the correct progression of the metabolic pathways in which this amino acid participates (remethylation and trans-sulphuration), the presence of cofactors is necessary. These cofactors include B vitamins, such as folic acid (vitamin B_9_), vitamin B_6_, vitamin B_2_, and vitamin B_12_. The reference range for the Hcy concentration is 5–15 μmol/L [44,45]. Excessively high plasma concentrations of Hcy, known as HHcy, lead to platelet activation and aggregation, hypercoagulability (activation of clotting factors V and VII), oxidative stress, vascular endothelial dysfunction, and the formation of atherosclerotic lesions. Hcy is considered an independent risk factor for arteriosclerosis [46,47,48].

Endothelial dysfunction is defined as an impairment in the vascular endothelium, which plays a critical role in maintaining homeostasis, including regulation of normal smooth muscle relaxation. The exact pathomechanism through which Hcy causes damage to endothelial cells is not fully understood. One hypothesis suggests that HHcy leads to increased oxidative stress in the vascular system [49]. The toxic effects of this amino acid may arise from the production of free radicals, which occurs when Hcy undergoes autooxidation to form Hcy disulfide or when it forms mixed disulphides with other thiols. The tissue factor (TF) is the main and primary initiator of the coagulation cascade. It is synthesised by monocytes, platelets, and endothelial cells in response to inflammatory factors, and it is a contributing factor to venous thrombosis. Hcy at physiological concentrations induces tissue factor expression in monocytes, which, in turn, exhibit dose- and time-dependent procoagulant activity in response to Hcy, even at Hcy concentrations of 10 μmol/L [50].

Hcy affects the cellular oxidative balance. The effect of Hcy on the activity of antioxidant enzymes in the cell appears to be dependent on the duration of exposure to elevated levels of Hcy, as well as the type of tissue exposed. In the case of the central nervous system (CNS), chronic HHcy (250 mg/kg body weight for 60 days) in mice was associated with increased activity of the cytosolic form of superoxide dismutase (Cu/ZnSOD) and catalase in the nucleus of the caudate shell and black nucleus. Similar results were obtained in a rat model, in which chronic HHcy (0.03 µmol/g body weight, twice daily for 30 days) increased superoxide dismutase (SOD) activity in the amygdala, catalase in the prefrontal cortex, and glutathione peroxidase (GPX) in both structures [51]. However, there were no significant changes in mitochondrial superoxide dismutase (MnSOD, SOD2) activity in the prefrontal cortex or hippocampus of rats treated with Hcy (0.03 µmol/g body weight, twice daily for 30 days) [51].

In the context of the cardiovascular system, there are apparent effects regarding the antioxidant activity of cells. Elevated oxidative stress in the vascular system has an inhibitory impact on two crucial antioxidant enzymes, glutathione peroxidase (GPX-1) and SOD. Hcy decreases the expression and activity of GPX-1. A study showed that Hcy-treated aortic endothelial cells had reduced GPX-1 activity (up to 81% at 250 mM of Hcy) [47]. Extracellular superoxide dismutase (EC-SOD) functions as a major ‘‘scavenger’’ of free radicals in the extracellular space. Human fibroblast production of EC-SOD was significantly inhibited at high concentrations of Hcy (1 mM), which was not observed at low concentrations (10 and 100 µM). No morphological changes or cytotoxic effects of Hcy were observed in human aortic endothelial cells or bovine aortic endothelium after 24 h of incubation in solutions with different concentrations of Hcy (0; 0.01; 0.1 and 1 mM). However, it was shown that Hcy-treated endothelial cells lost their ability to bind to EC-SOD, resulting in a loss of the protective capacity of the endothelium against oxidative stress [52]. A study showed an 88% decrease in MnSOD activity in rat myocardiocytes after a three-week supply of Hcy (0.3 mmol Hcy/g body weight in the first week; 0.4 mmol Hcy/g body weight in the second week; 0.6 mmol Hcy/g body weight in the third week) compared to the control group [53]. Similarly, after Hcy treatment of rat myocardiocyte cultures (30 μmol/L Hcy for 60 min), a significant decrease in catalase and SOD activity was observed. However, at the same time, GPX activity increased significantly [54]. The time-dependent effect of Hcy on GPX production was supported by a study involving both an in vivo rat model of HHcy (1.8 g/L of DL-Hcy for 4 weeks) and an in vitro model using a cell line of HUVECs (200 μM Hcy for 8h). Although cells in culture subjected to acute exposure to Hcy showed a statistically significant increase in GPX expression, mice with chronic HHcy were characterised by lower expression of the enzyme [55]. This indicates a time-dependent effect of HHcy on cells. 

Navneet et al. [56] showed that the effect of HHcy on antioxidant enzyme expression is dependent on the Keap1–Nrf2 pathway. In an in vitro model, they used Müller glial cells derived from the retina. Acute exposure to homocysteine (50 μM–1 mM for 24 h) led to activation of the Nrf2 pathway (with a peak expression of Nrf2 3 h after Hcy administration), increased expression of genes encoding antioxidant enzymes, including NQO1, CAT, SOD2, HMOX1, and GPX1, and a concomitant decrease in ROS and alleviation of oxidative stress [56]. Müller cells are the main glial cells in the retina, and they act as a support and protection for adjacent neurones. Importantly, a study showed that the different cells comprising the retina respond differently to excess Hcy—retinal ganglion cells in isolation show marked sensitivity and reduced viability, while glial cells in isolation show a strong antioxidant response combined with sustained viability over a wide range of Hcy concentrations [56]. 

Furthermore, using mouse Müller glial cells bearing Nrf2^−/−^ mutations, it was shown that Hcy affects the activation of antioxidant enzyme expression through the Keap1–Nrf2 pathway. Under physiological conditions, Keap1 (Kelch-like ECH-associated protein 1) binds to Nrf2 (Nuclear factor erythroid related factor 2) and promotes its degradation, which maintains low Nrf2 activity. However, when the Keap1 protein undergoes oxidative modification, this can trigger the release of Nrf2 from Keap1, consequently activating the Nrf2–Keap1 pathway. Hcy is one of the factors that can affect the oxidative modification of the Keap1 protein. Nrf2 in its free state is translocated to the nucleus, where it influences the expression of more than 500 genes related to antioxidant protection or cytoprotection [56]. Müller glial cells of mice carrying mutations in the Nrf2 gene were characterised by reduced viability and increased markers of oxidative stress compared to the wild type [56]. 

Sulphur-containing amino acids, such as Hcy, can undergo spontaneous processes that lead to the generation of free radicals. It is possible that the Hcy molecule is a source of intracellular superoxide anion (O^2−^). However, the inhibitory effect of Hcy on endothelial smooth muscle relaxation is probably due to increased O^2−^ production by endothelial cells [57]. To evaluate the effect that Hcy has on endothelium-dependent and independent vascular smooth muscle relaxation, Derek et al. used rabbit aortas. In addition, they assessed O^2−^ production by cultured porcine aortic endothelial cells. They also measured SOD activity in cell lysate from aortic tissue. Hcy was observed to significantly affect the concentration- and time-dependent inhibition of endothelium-dependent relaxation in response to acetylcholine and the calcium ionophore A23187. Incubation of porcine aortic endothelial cells with Hcy also caused a significant time-dependent increase in the concentration of O^2−^ released by these cells. Changes in O^2−^ concentrations were associated with a time-dependent increase in SOD activity in endothelial cells, which became significant after 72 h. Increased SOD activity may be considered a protective mechanism against oxidative stress in response to increased O^2−^ production after exposure to Hcy. Furthermore, the increase in O^2−^ in endothelial cells was completely inhibited by simultaneous incubation with Tiron (10 mmol/L), vitamin C (10 mmol/L), or vitamin E (10 mmol/L). However, the source of O^2−^ is unknown, as many cellular processes generate O^2−^ (including xanthine oxidase, NADH and NADPH oxidases, eicosanoid metabolism, and respiratory chain enzymes) [58]. Current data on the effects of HHcy on antioxidant protection are controversial. It has been suggested that there are different mechanisms that depend on variables, such as Hcy concentration or duration of the HHcy state, according to which Hcy affects the Keap1–Nrf2 pathway, thus leading to activation, or inhibition, of enzymes involved in protection against ROS. It is suggested that at least during the early stages of HHcy, Nrf2 factor-related antioxidant protection is activated.

**Figure 3:**
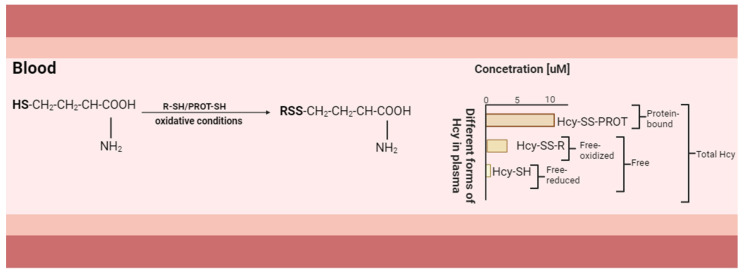
Forms of homocysteine (Hcy) with a special focus on blood oxidation. PROT-SH, Plasma protein containing thiol, mainly albumin; R-SH, Hcy, Cys, glutathione, glutamylcysteine, cysteinylglycine; PROT-SS-Hcy, Protein-bound Hcy; R, thiol or disulphide group, R-SS-Hcy, free, oxidised Hcy; HS-Hcy, free, reduced Hcy.

In the blood, where oxidative conditions prevail, Hcy, due to the increased reactivity of the thiol group, assumes oxidised states, accounting for approximately 98%, and at the same time may participate in a series of disulfide exchange reactions with various available thiol or disulphide compounds predominantly. Within this oxidised fraction, about 75% forms complexes with proteins (Hcy-SS-PROT), primarily with albumin. The remaining fraction is represented by nonprotein-binding disulfides, which include Hcy, homocysteine–cysteine disulphide, and lesser amounts of other mixed disulfides, such as homocysteine–cysteinylglycine disulfide (described as Hcy-SS-R) [59]. Only a small percentage, approximately 1–2%, adopts the reduced form, which is characterised by a free thiol group (Hcy-SH) [60] (Figure 3).

Depending on the plasma concentration of Hcy, HHcy can be divided into moderate, intermediate, and severe forms. In the most severe form (concentrations above 100 μmol/L), there is a significant increase in the risk of thromboembolic complications, which contribute to increased mortality. According to the American Hospital Association (AHA), the normal range of Hcy concentrations is between 5 and 15 μmol/L. However, some sources consider concentrations above 12 μmol/L to be elevated. In 2006, the American Stroke Association and the American Society of Hypertension adopted Hcy concentrations above 10 μmol/L as criteria for HHcy [61]. Intermediate HHcy occurs when its plasma concentration is in the range of 30–100 μmol/L. Severe HHcy is a condition in which the Hcy concentration exceeds 100 μmol/L, primarily due to an inborn disorder in Hcy metabolism resulting in the development of homocystinuria. Homocystinuria, first described by Mudd et al., is associated with skeletal muscle weakness, seizures, and abnormal encephalographic recordings. Biochemical studies have observed normal Met levels, normal CBS activity in fibroblasts, and decreased MTHFR activity [11]. 

The NATPOL study shows that in Poland, HHcy occurs in 17% of adults. However, in people over 59 years of age with a high risk of ischemic stroke (IS), the prevalence of HHcy exceeds 29% [62]. Numerous genetic and environmental factors cause HHcy. Among the leading factors, we distinguish CBS deficiency, MTHFR deficiency, and vitamin B_12_, folic acid, or vitamin B_6_ deficiency. Numerous studies have been published demonstrating the correlation between plasma Hcy concentrations and IHD or venous thromboembolism. According to Seo et al. [63], every 5 µM/L increase in Hcy concentration increases the risk of CVD by 50% and also increases total cholesterol levels by 20 mg/dL. The occasionally inconclusive results can be attributed to a variety of factors that influence the concentration of this amino acid, including diet habits, genetic conditions, and the racial composition of the studied population.

Several mechanisms are known to underlie the basis for CVD development. In response to increased plasma Hcy concentrations, there may be vascular endothelial dysfunction, reduced expression of thrombomodulin and activated protein C [64], a decrease in the presence of anticoagulant heparans [65], and a decrease in tissue plasminogen activator receptors on the surface of vascular endothelial cells [66]. 

Homocysteine affects the functioning of blood vessels as well as the viability of nerve cells [67]. This may be especially pertinent in cases of concurrent type 2 diabetes mellitus (T2DM) and atherosclerosis or coronary artery disease. The occurrence of elevated levels of Hcy in individuals with T2DM is associated with a greater incidence of macroangiopathy and nephropathy [68]. 

Elevated Hcy levels result in various detrimental effects on both vascular and immune cells. These effects are mainly triggered by the generation of reactive oxygen species (ROS) and encompass endothelial damage, platelet activation, smooth muscle cells, LDL oxidation, and the facilitation of interactions between endothelial cells and monocytes. Additionally, similar to interleukin-6 (IL-6) or high glucose concentrations, Hcy induces the formation of neutrophil extracellular traps (NETs). This suggests that Hcy may impact immune function by promoting NETosis and disrupting platelet and neutrophils, thus potentially increasing the risk of thromboembolic complications, especially in patients with T2DM [69]. Hcy undergoes oxidation to disulphides (R-SS-Hcy) under aerobic conditions and at physiological pH [70]. In addition, a small fraction of Hcy is converted by aminoacyl-tRNA synthetase to a cyclic form, homocysteine thiolactone (HCTL) [71]. Hcy can modify proteins by binding to thiol groups in a process named S-homocysteinylation. Hcy may also form permanent amide bonds in reactions between the activated carbonyl group of HCTL and the ε-amino group of lysine residues (N-homocysteinylation). HCTL is unstable and can be hydrolysed back to Hcy. Under physiological pH conditions, 1 mM of HCTL hydrolyses to ~0.71 mM of Hcy within 24 h. Incubation of 1 mM of HCTL with albumin demonstrates that the S homocysteinylation reaction predominates over the N homocysteinylation reaction of albumin [72].

**Figure 4:**
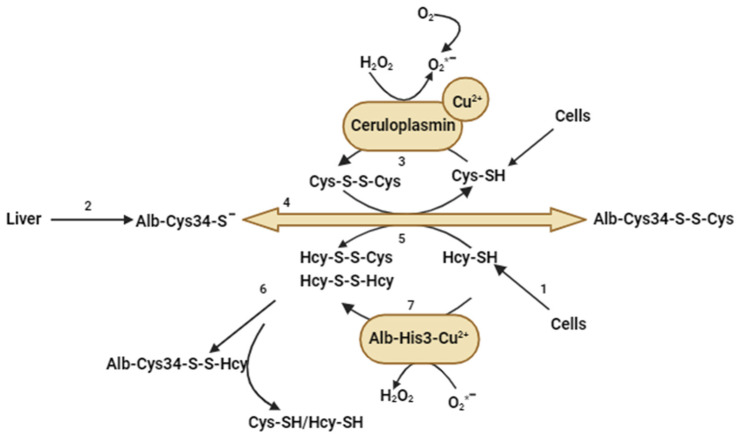
Formation of homocysteine (Hcy) and albumin-bound Hcy in circulation. 1—Transport of free reduced homocysteine (Hcy-SH) from cells to the circulation. 2—Hepatic secretion of albumin thiolate anion (Alb-Cys34-S^−^) into the circulation. 3—Auto-oxidation of free reduced cysteine (Cys-SH) to cystine (Cys-S-S-Cys) by ceruloplasmin. 4—Reaction of the albumin thiolate anion (Alb-Cys34-S^−^) with cystine (Cys-S-S-Cys) to form the Alb-Cys34-S-S-Cys and cysteine thiolate anion. 5—Reaction of free reduced homocysteine (Hcy-SH) with cysteine-bound albumin (Alb-Cys34-S-S-Cys) to form mixed homocysteine–cysteine disulphide (Hcy-S-S-Cys) and albumin thiolate anion (Alb-Cys34-S^−^) 6—Reaction of albumin thiolate anion with Hcy-S-S-Cys and with homocystine (Hcy-S-S-Hcy). 7—Auto-oxidation of Hcy to homocystine (Hcy-S-S-Hcy) through reaction with copper-His3 of albumin; O_2_*^−^, superoxide anion; *, free radicals form.

Albumin has a single cysteine residue at position 34 (Cys 34). In the circulation, the majority of Cys 34 (>65%) is present in the reduced free form (albumin-Cys34-SH), while one third of albumin-Cys34 is bound to cystine (albumin-Cys34-SS-Cys) and approximately 2% is bound to Hcy (Figure 3). In the circulation, free cysteine (Cys-SH) undergoes oxidation to form cystine (Cys-S-S-Cys) in a process mediated by ceruloplasmin (Figure 4). The superoxide radical readily reacts with Cu^2+^, which serves as a cofactor for ceruloplasmin. This reaction leads to the generation of H_2_O_2_ and consequently alters the oxidation reduction potential of the cell [73]. Albumin plays an important role in the oxidation of Hcy. The albumin thiolate anion (Alb-Cys34-S-) is the form secreted into the blood by the liver. It reacts with cystine (Cys-S-S-Cys). Hcy then binds to the Cys 34 and forms albumin’s thiolate anion and a mixed Hcy–cysteine disulphide. In the second step, the albumin thiolate anion reacts primarily with the mixed Hcy–cysteine disulphide, resulting in a product in which Hcy is mainly bound to albumin (Alb-Cys34-S-S-Hcy) (Figure 4) [74]. In albumin-deficient plasma, we observed a reduction in the conversion of Hcy to its disulphide form, indicating the paramount role of albumin availability in these reactions [72]. Studies have shown that homocysteinylated albumin induces an inflammatory response, increases monocyte adhesion to endothelial cells [75], and may contribute to cell apoptosis [76].

**Figure 5:**
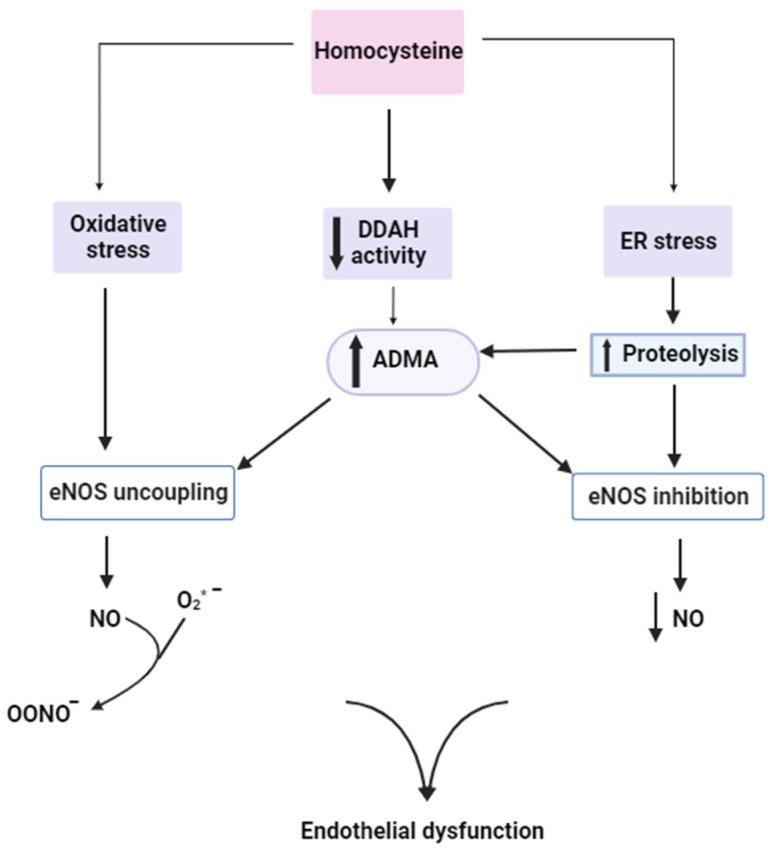
Processes leading to endothelial dysfunction induced by homocysteine and asymmetric dimethylarginine (ADMA). O_2_*^−^, superoxide anion; OONO^−^, peroxynitrite anion.

Endothelial cells produce nitric oxide (NO) in a reaction catalysed by endothelial nitric oxide synthase (eNOS). NO has a vasodilatory effect on blood vessels, helps to maintain vascular wall tension, and inhibits platelet aggregation and proliferation of the vascular smooth muscle [77,78]. In the endothelium, folates serve as donors of methyl groups, as well as donors of hydrogen and electrons necessary for the reduction of dihydrobiopterin (BH_2_) to tetrahydrobiopterin (BH_4_). BH_4_ is a cofactor of eNOS. The probable reason for the uncoupling of eNOS, which is marked by a mismatch between eNOS levels and NO production, is a decreased concentration of BH_4_. Under normal conditions, two BH_4_ molecules bind to each eNOS subunit, thereby facilitating electron transfer for the oxidation of L-arginine. When BH_4_ concentrations decrease, eNOS generates ROS that intensify oxidative stress. At the same time, NO reacts with the superoxide ion (O^2−^) to form the peroxynitrite ion (ONOO^−^), consequently reducing the availability of NO. Therefore, the presence of BH_4_ is crucial for proper endothelial function [79]. However, the mechanisms through which HHcy reduces the bioavailability of endothelial NO are multifactorial. Asymmetric dimethylarginine (ADMA), an endogenous inhibitor of eNOS, may play a pivotal role in this process. Hcy can increase ADMA concentrations by inhibiting the activity of dimethylaminohydrolase (DDAH), which is responsible for decomposition of ADMA. Hcy exerts its inhibitory effect on DDAH either directly by reacting with its cysteine components or indirectly by inducing oxidative stress, which leads to the oxidative inactivation of DDAH. Furthermore, Hcy can raise ADMA levels by inducing endoplasmic reticulum (ER) stress and apoptosis, resulting in increased proteolysis of proteins containing methylarginine residues. The accumulation of ADMA in vascular endothelial cells inhibits eNOS, leading to decreased NO production. Elevated concentrations of ADMA also affect NO inactivation by uncoupling eNOS and increasing the superoxide anion radical of NO (Figure 5) [80]. Additionally, Hcy can reduce NO bioavailability by affecting the function of antioxidant enzymes, such as SOD, catalase, and GPX [81].

Normal endothelial cells modulate the effect of Hcy on NO concentration by generating S-nitrosothiols. In this reaction, highly reactive nitrosyl ion NO^+^ is attached to -SH groups, resulting in the formation of a nitrosothiol group (SNO) [82].

In vitro studies conducted on endothelial cells isolated from the bovine aorta indicate that prolonged exposure (>3 h) to Hcy results in a compromised response to endothelium-derived relaxing factor (EDRF). On the contrary, a brief (15 min) exposure stimulates EDRF secretion, resulting in the production of S-NO-Hcy, a potent antiplatelet agent and vasodilator. In contrast to Hcy, S-NO-Hcy does not stimulate the production of H_2_O_2_ and does not undergo transformation into HCTL, which is considered a toxic substance to the endothelium. These results imply that a properly functioning endothelium controls the potential harmful effects of Hcy through the release of EDRF and the formation of S-NO-Hcy. The adverse vascular effects of Hcy may result from a vascular inability to maintain nitrosothiol formation due to an ongoing imbalance between NO production and Hcy levels. This leads to progressive endothelial dysfunction [83]. 

## 5. *MTHFR* Gene Variants with Hyperhomocysteinemia and Cardiovascular Diseases

Elevated Hcy levels are considered an independent risk factor for the development of CVD, which encompasses CAD, MI, IS, hypertension, and thrombosis [84,85].

HHcy may result from a deficiency in essential dietary components, such as vitamin B_6_, B_12_, and folate. These vitamins act as donors of methyl groups in the remethylation reaction. Additionally, HHcy can be genetically determined. The *MTHFR* gene, which encodes the pivotal enzyme in folate processing, exhibits various common or rare single nucleotide polymorphisms (SNPs) that can impact the activity of the MTHFR enzyme. In particular, among these variants, rs1801133 (C677T) and rs1801131 (A1298C) are commonly identified and associated with reduced MTHFR activity. 

In individuals carrying the C677T variant, enzyme activity decreased to approximately 67% and 25% for those with one copy (heterozygous) and two copies (homozygous) of the T allele (*MTHFR* C677T polymorphism), respectively. Regarding the variant A1298C, those with one copy and two copies of the allele show enzyme activity levels of approximately 83% and 61%, respectively, compared to individuals with the wild-type variant [86]. Furthermore, individuals with the 677TT genotype show an increased thermolability of the MTHFR enzyme and elevated plasma Hcy concentrations [6].

Folate deficiency significantly contributes to the manifestation of HHcy in individuals with the 677T allele. Research has shown that in individuals with lower plasma folate concentrations (<15.4 nmol/L), those with the homozygous mutant genotype had total fasting Hcy levels 24% higher than those with the 677CC genotype. However, there were no significant differences between genotypes among individuals with folate levels ≥ 15.4 nmol/L [85]. 

A report from the AHA (American Heart Association) reveals that CVD is the leading cause of death worldwide [87]. CVD includes various conditions, such as MI. Two meta-analyses have investigated the association between the C677T polymorphism and an increased risk of MI. The first meta-analysis, conducted in 2011, summarised the results of 30 studies involving a total of 8140 patients and 10,522 controls. The *MTHFR* C677T polymorphism was found to influence the risk of MI in young or middle-aged Caucasians (under 50 years of age). Individuals with the TT genotype had a higher risk of MI than heterozygotes with the CT genotype (OR = 1.275). However, no similar correlation was observed in older people or among Asians or African Americans [88]. 

Another meta-analysis conducted in 2016 by Li et al. [89] included 44 studies, which collected data from a total of 9693 cases and 12,554 controls of both Caucasian and Asian races. This analysis found no association between the C677T polymorphism and the risk of MI in the entire group or in subgroups that considered ethnicity and gender [89].

The main risk factors for CAD include dyslipidaemia, T2DM, obesity, smoking, diet, gender, and genetic variation [90]. In recent years, there has been a growing interest in studying the role of genetic variation in the development of CAD. The influence of the C677T polymorphism on the pathogenesis of this condition has also been analysed. In a meta-analysis among the Chinese population, which included 33 studies (6130 study patients and 6163 controls), it was found that the C677T polymorphism was associated with a higher risk of CAD (TT vs. CC: OR = 1.88) [91].

In addition, the groups were stratified by geographic location. Interestingly, the C677T polymorphism posed a higher risk factor for CAD in patients from northern China (TT vs. CC: OR = 1.92) compared to southern China (TT vs. CC: OR = 1.69) [91]. This difference is likely attributed to dietary folic acid deficiency, which is more prevalent among northern Chinese residents. These disparities were already demonstrated in a large population study in 2001 involving 2422 Chinese patients 35–64 years of age. Among them, approximately 40% of the people in the northern regions and only 6% of the people in the southern regions had plasma folate concentrations less than 6.8 nmol/L. Similarly, folate concentrations in red blood cells lower than 363 nmol/L were observed in approximately 30% of northerners and only 4% of southerners [92]. This suggests that insufficient dietary folic acid intake and impaired MTHFR activity may be significant risk factors for the development of CVD.

Cardiovascular disease also includes coronary heart disease (CHD). CHD is characterised by an imbalance between myocardial oxygen demand and supply, which results in hypoxia and heart failure. In 2002, an extensive cross-population meta-analysis involving 22 studies in the European population, 10 studies in the North American population, and 8 studies from Japan, Australia, Israel, and Turkey included a total of 11,162 patients with coronary heart disease. Individuals with the 677TT genotype had a 16% increased risk of developing CHD compared to CC homozygotes. Individuals with TT and CT genotypes, from both the study and the control groups, had higher plasma Hcy concentrations and lower folate concentrations compared to those with the CC genotype. This supports the hypothesis that impaired folate metabolism leading to high Hcy concentrations plays a crucial role in the pathophysiology of CHD. Upon geographic analysis, it was also observed that the 677TT genotype was closely associated with an increased risk of developing CHD in Europeans (OR = 1.14), while this association was not observed in the North American population (OR = 0.87). The increased risk of CHD in 677TT homozygotes was dependent on the presence of low serum folic acid levels. The difference between the European and American populations can be attributed to the enrichment of foods with folic acid in North America. Therefore, folic acid deficiency was not observed in North America, so the effect of the C677T *MTHFR* polymorphism did not influence the risk of developing CHD [93].

In the Copenhagen City Heart Study, *MTHFR* genotyping was performed in 9238 individuals from the Danish population. However, no association was found between the 677TT genotype and an increased risk of IHD and venous thromboembolism, even after considering 12 risk factors for their development. These factors included gender, age, total cholesterol, high-density lipoprotein (HDL) fraction cholesterol, triglycerides, fibrinogen, lipoprotein(a), factor V Leiden genotype, and also BMI, smoking, diabetes, and hypertension [94].

The synergistic effect of the *MTHFR* C677T polymorphism and environmental factors on the risk of hypertension was analysed among 708 Chinese Han patients with a mean age of 46 years. The frequency of the 677T allele in the hypertensive group was 60.77%, which was higher compared to patients with normal blood pressure (53.76%). Patients with CT and TT genotypes had a higher risk of hypertension (OR = 1.52). Incorporating both genotype and overweight/obesity into the analysis was associated with a significant increase in the risk of hypertension (OR = 3.90). Patients with the TT genotype who were overweight or obese exhibited elevated levels of total cholesterol and triglycerides, as well as reduced levels of HDL cholesterol. The interaction between C677T polymorphism, overweight/obesity, and dyslipidaemia may play a key role in the increased risk of hypertension [95].

In China, there are approximately 2.5 million new stroke cases per year, with over one million individuals experiencing stroke-related complications [96]. Epidemiological studies have also indicated an association between the *MTHFR* polymorphism and an increased risk of IS. In the Hakka population of southern China, the presence of the T allele was shown to be an independent risk factor for developing IS. The study included 1967 patients with IS and 2565 controls. A positive correlation was also observed in the dominant model, indicating that individuals with both the TT genotype and the CT genotype were more susceptible to developing IS compared to CC homozygotes. It is worth mentioning that there are certain limitations to that study, including the lack of information on folic acid supplementation, the absence of serum folic acid level assessments in patients, and the exclusive focus on a single genetic polymorphism [97].

In Poland, a study was conducted on the association of the C677T polymorphism with the risk of IS. The frequency of the TT genotype was significantly higher in the study group (n = 152) than in the control group (n = 135) (11.8% vs. 4.4%). In both men and women, the possession of the TT genotype was associated with an IS risk greater than two-fold (OR = 2.14) [97]. 

Different results were presented in an analysis by Q.-Q. Lv. et al. [98] in 2014, where the effect of the C677T and A1298C variants of the *MTHFR* gene on the incidence of IS was evaluated in the eastern Chinese population of 199 patients (study group) and 241 controls. Interestingly, the C variant of the A1298C polymorphism was observed more frequently in IS patients (60.1%) compared to the control group (47.7%), suggesting a strong association of the C allele with IS. CC homozygotes and AC heterozygotes of the A1298C polymorphism had a significantly higher risk of developing IS (OR = 2.55). Unlike previous analyses, no association was observed between the C677T polymorphism and IS in the study population [98]. 

However, studies on the effect of *MTHFR* genotypes on cardiovascular disease (CVD) risk often produce conflicting results. Discrepancies can be attributed to the multifactorial aetiology of CVD, small study group sizes, ethnic differences in the populations studied, and lack of consideration of the consumption of essential compounds within the MTHFR metabolic pathway, such as folic acid or vitamin B_12_. The interactions between environmental and genetic factors, including different variants of *MTHFR*, are important in explaining this variability. 

Due to the complex and multifactorial nature of CVD, ongoing research is essential to assess the significance of interactions between modifiable and nonmodifiable risk factors. Among the unmodifiable risk factors, we can distinguish genetic predisposition, race, gender, and ageing. Within the modifiable factors, we can differentiate clinical, environmental, behavioural, and psychosocial elements [99]. Modifiable factors, such as T2DM, hypertension, smoking, obesity, or air pollution, are generally considered more influential than genetic factors. In the context of genetic factors, the presence of gene–gene interactions should be taken into account, as well as the existence of other polymorphisms, such as the *NOS3* G894T, the *ACE I/D* polymorphism, or the *A1166C* polymorphism of the type 1 receptor for the angiotensin II gene.

Furthermore, the association between the C677T polymorphism of the *MTHFR* gene and CVD may be influenced by the presence of concomitant HHcy, which is acknowledged as a significant and independent risk factor for CVD. Unfortunately, there is no clear indication of the direction of these relationships. The example is a study involving 63 Brazilian patients aged 46 to 68 who received coronary angiography. The patients were classified according to the severity of atherosclerosis, including those without atherosclerosis, those with mild/moderate atherosclerosis, and those with acute atherosclerosis. Significant differences in Hcy concentrations in the group with acute atherosclerosis compared to the control group (17.0 ± 7.4 µmol/L vs. 10.6 ± 3.9 µmol/L) were observed. However, no correlation was found between the presence of the C677T variant and HHcy [100]. Deficiencies in folates have adverse effects on the one-carbon cycle, thus leading to the accumulation of Hcy and an imbalance between different form of folates, which might be a stronger risk factor than the presence of an unfavourable variant of C677T polymorphism.

## 6. The Association of Variants of the *MTHFR* Gene with Obesity and Accompanying Disorders of Lipid and Carbohydrate Metabolism

Obesity is a chronic disease characterised by an increase in body weight due to an excess accumulation of body fat. According to the World Health Organisation (WHO), obesity is characterised by a body mass index (BMI) equal to or exceeding 30 kg/m^2^. Currently, obesity is one of the most pressing global health issues, with increasing prevalence reaching epidemic proportions in both economically developed and developing countries. Since 1975, obesity has nearly tripled, and in 2016, there were more than 650 million obese adults worldwide, comprising 13% of the global population. The World Obesity Federation estimates that by 2025, a quarter of the world’s population will suffer from obesity. Particularly alarming are the data indicating an increase in average BMI and the prevalence of obesity among children and adolescents aged 5–19 in most countries. In 2016, approximately 6% of girls and 8% of boys were estimated to be classified as obese. 

The prevalence of obesity in the United States has consistently remained higher than in other countries. The age-adjusted prevalence of obesity in adults was 42.4%, while the age-adjusted prevalence of severe obesity (BMI < 40) in adults was 9.2% in 2017–2018. The overall prevalence of obesity was similar among men and women, but the prevalence of severe obesity was higher among women (11.5% vs. 6.9%). Furthermore, adults aged 40 to 59 years had the highest prevalence of severe obesity (11.5%) [101]. 

Obesity is a complex disease. In addition to environmental factors, such as a high-energy diet and low physical activity, there is a significant genetic influence. The high variability between individuals in response to environmental factors indicates a genetic predisposition to excessive fat accumulation [102]. Currently, the genetic basis of obesity and obesity-related diseases is an area of interest for numerous research centres. Understanding the genetic basis of obesity is considered a crucial factor that will contribute to enhancing the effectiveness of preventive and therapeutic measures.

Genes associated with obesity development include the *MTHFR* gene. The 677T allele has been associated with an increased risk of overweight and obesity, as well as associated diseases, which include dyslipidaemia or insulin resistance. Unfortunately, the results of studies are frequently inconclusive.

One of the larger studies that focused on the relationship between obesity and C677T polymorphism came from three large, population-based cohorts: the British Women’s Heart and Health Study (BWHHS), The Avon Longitudinal Study of Parents and Children, and the Copenhagen City Heart Study, with a total of 24,210 patients. A positive association between the TT genotype and obesity (BMI > 30 kg/m^2^) was found only in patients with BWHHS [103]. 

Similarly, no statistically significant differences were found in the prevalence of the *MTHFR* C677T polymorphism between obese and non-obese individuals in 1712 individuals of Polish origin [104], in a young population in Mexico [105], or in a population of Brazilian patients [106]. Regarding the first of the mentioned studies, a correlation was established between excessive body weight, the TT genotype of the *MTHFR* gene, and reduced folate levels. The study also demonstrated the influence of radical weight loss on folate concentrations. In individuals with obesity and the TT genotype, serum folate levels were markedly lower (6.6 ± 2.9 ng/mL) compared to those with the CT (8.0 ± 3.7 ng/mL) and CC (8.2 ± 3.7 ng/mL) genotypes. 

The complications resulting from overweight and obesity depend essentially on the distribution of body fat and the severity of the condition. Obesity underlies many chronic diseases, such as T2DM, CVD, hypertension, obstructive sleep apnoea, and cancer. It also leads to the development of insulin resistance and abnormal glucose and lipid metabolism [107], as well as the secretion of numerous pro-inflammatory cytokines [108] and procoagulant factors [109]. As mentioned above, the association between the *MTHFR* 677C>T polymorphism and overweight/obesity, as well as lipid profiles, glucose levels, and waist circumference, was studied in a young Mexican population of 316 individuals (172 normal weight, 74 overweight, and 70 obese patients). No differences were observed in the distribution of genotypes between normal-weight and overweight or obese individuals. Furthermore, after dividing the study group by gender, it was surprising that the TT genotype appeared to have a protective effect against the development of obesity in the male study group, while women with the TT genotype had a smaller waist circumference [105]. 

The gold standard for body composition testing is the Dual-energy X-ray absorptiometry (DXA) study. The DXA and anthropometric studies were carried out in 56 obese Italians who followed a low-calorie Mediterranean diet for 12 weeks. The aim was to assess whether the C677T polymorphism has an influence on achieving greater weight loss during weight loss therapy in patients and whether the genotype can contribute to maintaining the effects of this therapy over a longer period. The frequency of the T allele in the study group was 19.6%, and the genotype frequencies of the CC, CT, and TT individuals were 69.6% (n = 39), 21.4% (n = 12), and 8.9% (n =5), respectively. Before the dietary intervention, people carrying the T allele exhibited significantly higher body weight, BMI, and waist, abdominal, and hip circumference, as well as a higher waist/hip ratio and greater total body fat mass. The genotype analysis revealed an association between C677T polymorphism and body composition variables both before and after the 12-week intervention involving the Mediterranean diet. Following the diet, there was a difference in the ratio of total lean body mass (LBM), which mainly comprises muscle mass, to total fat mass between the different genotypes. This indicated that carriers of the T allele primarily lost muscle mass. For these individuals, it is important to pay special attention to their dietary protein intake, as it can play a vital role in both enhancing and maintaining lean body mass. The authors suggest that personalising dietary interventions based on the *MTHFR* genotype could be considered. The *MTHFR* gene is highly expressed in skeletal muscle because muscle formation is associated with the simultaneous production of Hcy in relation to creatine/creatinine synthesis. The gender–genotype interaction observed in men is likely due to their greater muscle mass [110,111].

It is well known that a higher birth weight is associated with a higher BMI in childhood and later life, although the relationship is more intricate. Higher birth weight is associated with higher fat-free mass in later life than with higher body fat mass. Frelut et al. [112] investigated whether there was a correlation between C677T polymorphism, birth weight, and the risk of developing CVD in 113 morbidly obese French adolescents (BMI = 39.1 ± 6.4 kg/m^2^) at the age of 14.4 ± 1.5 years. The birth weight was lower in those with the TT (2.95 ± 0.48 kg) compared to those with the CC (3.34 ± 0.43 kg) and CT (3.38 ± 0.50 kg) genotypes. This pattern persisted throughout the first year of life. At 2 years of age, children with the TT genotype achieved BMIs similar to those with other genotypes. In this group, the onset of obesity was preceded by an early growth phase and rapid weight gain. Furthermore, the basal mean insulin was significantly higher in TT homozygotes (30.5 ± 21.6 UI/mL) than in individuals with CC (18.5 ± 9.8 UI/mL) or CT (19.0 ± 10.2 UI/mL). The median fasting insulin concentrations and HOMA IR were 58% and 64% higher, respectively, in TT patients compared to individuals with CC or CT. This suggests that the C677T polymorphism is related to early growth and the development of insulin resistance in morbidly obese individuals, with the T allele being the responsible factor [112].

The results of that experiment are confirmed by an analysis by Ong KK. He showed that low birth weight and subsequent rapid weight gain are factors that lead to childhood obesity and, later, to insulin resistance. This may be due to the fact that lower birth weight is associated with a later higher ratio of fat mass to fat-free mass and more abdominal adipose tissue [113].

The aforementioned reports demonstrate the significant role of the *MTHFR* gene polymorphism in lipid metabolism and its effects on BMI. Obesity represents a substantial risk factor for the emergence of CVD, as it leads to the earlier onset of CVD events and a shorter average lifespan compared to people of normal weight [114]. The potential involvement of the C677T variant in the pathogenesis of CVD, including CHD, is primarily associated with increased Hcy levels in most studies. HHcy and subsequent hypomethionemia are independent risk factors for endothelial damage through the excessive proliferation of smooth muscle cells, H_2_O_2_ production, as well as protein and lipid oxidation [115]. High intake of saturated fatty acids (SFAs) was detected to be associated with high plasma Hcy concentrations. To explain this, a study involving 5917 individuals from the Norwegian population divided into two age groups (47–49 and 71–74 years) was conducted using food frequency questionnaires and measurements of plasma total homocysteine (tHcy) concentrations. The increased intake of SFAs is correlated with higher concentrations of tHcy in plasma. The difference in plasma tHcy between the extreme quartiles of SFA intake was 8.8%. However, a diet rich in long-chain omega-3 fatty acids appeared to reduce Hcy concentrations only with high B vitamin supplementation [116].

Among the Turkish population, there was an association between the C677T polymorphism and BMI in patients with CHD, including 82 with diabetes and 112 without diabetes. In one study, it was revealed that among diabetic patients with CHD, the frequency of obesity was found to be higher in the CC genotype (27.84 ± 4.24) than among those carrying the T allele (27.84 ± 4.24 in CC, 24.09 ± 3.38 in TT). Typically, lipids tend to exert a positive influence on BMI. However, examination of the correlation between BMI and *MTHFR* genotypes according to sex revealed that in diabetic women, the wild-type genotype (CC) elevated BMI independently of lipids (*p* = 0.009). However, this association was not evident in males. Furthermore, insulin resistance seems to elevate Hcy levels. Consequently, the observed influence of the *MTHFR* polymorphism on BMI in patients with diabetes and CHD might be attributed to elevated insulin levels. Furthermore, other genetic variations within the *MTHFR* gene, such as A1298C, could have exerted an additional influence [117]. 

Obesity is an important risk factor for type 2 diabetes mellitus (T2DM). Women with a BMI of 30 kg/m^2^ have a 28 times higher risk of developing T2DM compared to women of normal weight. Furthermore, a BMI of 35 kg/m^2^ increases the risk of T2DM by 93 times [118]. The interactions between the prevalence of overweight and obesity, variants of the *MTHFR* gene, and the risk of developing T2DM are not fully understood. Studies have revealed that HHcy can induce insulin resistance in adipose tissue by inducing ER stress through activation of c-Jun N-terminal kinase (JNK) [119]. Hyperactivation of JNK leads to serine phosphorylation in IRS-1 (insulin receptor substrate-1) and inhibition of insulin receptor signaling [120]. A study conducted on rat adipocytes and mice with HHcy showed that HHcy impairs glucose transport, particularly the insulin signaling pathway. This impairment occurs through the reduction of insulin-stimulated tyrosine phosphorylation of the insulin receptor and IRS-1, an increase in serine phosphorylation of IRS-1, and, hence, inhibition of Akt phosphorylation. These impairments were accompanied by an increase in resistin expression [121]. Resistin, an adipokine produced by mature adipocytes and macrophages, serves as a key mediator of insulin resistance and is closely related to the generation of pro-inflammatory cytokines by adipocytes via the ROS–PKC–NF–ĸB pathway [121]. Furthermore, resistin is associated with the inflammatory environment due to its primary secretion by monocytes and its correlation with IL-6 levels [122].

The relationship between the C677T polymorphism and insulin resistance was investigated among 132 patients who had been diagnosed with insulin resistance. Measurements of blood pressure, BMI, and waist and hip circumference were made, and fasting blood glucose, insulin levels, and lipid parameters were also tested. The frequency of CT and TT genotypes was significantly higher in patients with insulin resistance compared to controls (OR = 1.68). The C677T allele was also associated with obesity, hypertriglyceridemia, and low levels of HDL fraction cholesterol [123]. 

Nurses are particularly susceptible to developing insulin resistance due to the adverse effects of shift work, which disrupts the regulation of the biological clock. The biological clock is an internal oscillator comprising biochemical processes occurring in the body’s cells and tissues regulated by gene products independently of changes in the external environment. A meta-analysis conducted in 2023 demonstrated that shift work, characterised by disrupted sleep–wake rhythms and disrupted eating patterns, has altered the functioning of the biological clock. This, in turn, results in changes in metabolic parameters, such as glucose, insulin, and cholesterol levels [124]. Physiologically, the body exhibits diurnal variability in glucose tolerance and insulin resistance. Glucose tolerance is greatest in the morning, while insulin resistance is greatest in the evening. Thus, the disrupted meal intake pattern often observed in shift workers is a risk factor for the development of insulin resistance and T2DM [124]. The association between *MTHFR* 677C>T polymorphism and insulin resistance was studied among 273 nurses aged 22–57 years working in five hospitals at the Tehran University of Medical Sciences. The frequency of genotypes was CC—9.2%, CT—51.6%, and TT—35.2%. An association was observed between the polymorphisms studied and the HOMA-IR index and serum insulin levels. Nurses with variant T had a significantly higher HOMA-IR index compared to CC homozygotes (*p* = 0.03). Participants carrying the TT genotype (9.65 ± 4.00 µU/mL) exhibited significantly lower insulin levels compared to participants with the CT genotype (14.12 ± 15.34 µU/mL). The effect of taking folic acid and vitamin B_12_ on HOMA-IR was also investigated. This index was shown to be lower in women taking supplements regardless of their genotype [125].

The association of *MTHFR* C677T and *MTRR* A66G polymorphisms with the development of T2DM was studied among a Chinese Han population, including 180 patients with T2DM and 350 healthy controls. It was investigated that the *MTHFR* C677T (OR = 1.78 in the homozygous codominant genetic model) and *MTRR* A66G (OR = 1.43 in the dominant genetic model) were associated with a higher risk of T2DM [126]. The distributions of the C677T variants were also compared among 682 Iranian patients with different levels of obesity. The patients were divided into four groups: normal weight, obese, with T2DM and obese, and with T2DM. Individuals with the TT genotype exhibited significantly elevated plasma Hcy levels (34.6 ± 26.5 µmol/L) compared to those with the CC genotype (15.1 ± 8 µmol/L) or the CT genotype (16.4 ± 7.8 µmol/L). However, unlike previous studies, there were no significant differences in genotype frequencies among the groups [46]. 

Metabolic syndrome (MetS) is a complex disorder that involves the presence of obesity and two of the three following criteria: high blood pressure, impaired glucose metabolism, and elevated non-high-density lipoprotein (non-HDL) cholesterol level (atherogenic dyslipidaemia) [127]. The presence of these metabolic abnormalities increases the risk of developing atherosclerotic cardiovascular disease and T2DM. Researchers do not agree on the clear aetiology of MetS. Insulin resistance and central obesity are regarded as the primary causes. Genetic predispositions are also an important risk factor [128].

In the Asian population, 158 patients with T2DM (118 with MetS and 40 without MetS) and 55 healthy individuals were analysed. Serum insulin, folic acid, and vitamin B_12_ concentrations were assessed through radioimmunoassay, while hs-CRP concentrations were measured through turbidimetric assay. Individuals with the TT genotype had significantly higher concentrations of triglycerides (2.46 ± 2.04 mmol/L—TT genotype, 2.63 ± 1.75 mmol/L—CT genotype, 1.74 ± 1.07 mmol/L—CC genotype) and CRP (3.17 ± 1. 82 mg/L—CC genotype, 4.43 ± 3.09 mg/L—CT genotype, 5.54 ± 4.25 mg/L—TT genotype) and a higher HOMA-IR index (1.95 ± 1.24 CC genotype, 2.69 ± 0.88 CT genotype, 3.73 ± 0.84 TT genotype) compared to CC homozygotes and CT heterozygotes. Serum vitamin B_12_ levels in individuals with the TT genotype (388.42 ± 213.20 pg/mL) were lower than in patients with the CC genotype (599.73 ± 265.99 pg/mL) or the CT genotype (498.25 ± 235.66 pg/mL). The interaction between adverse variants of the *MTHFR* gene and increased CRP levels and decreased vitamin B_12_ levels may explain the increased predisposition of these patients to developing insulin resistance [129]. 

A group of 58 people with psychotic disorders taking neuroleptics for 12 months was subjected to assessments of MetS, insulin resistance, and *MTHFR* C677T and A1298C polymorphisms. Among individuals carrying the 677T allele, 53% met the MetS criteria, whereas those with the CC genotype had a lower percentage of 23% (OR = 3.7). Patients with the T allele had a risk nearly four times higher, despite similar exposure to antipsychotic drugs. Both waist circumference and the TT genotype showed significant correlations with insulin resistance, indicating that these individuals are at increased risk of developing insulin resistance [130]. 

Comparable findings were achieved at Renmin Hospital of Wuhan University, where a study involved 651 people diagnosed with MetS and 727 healthy individuals. People with the TT genotype of the *MTHFR* gene were shown to have a higher risk of developing MetS (*p* = 1.59) compared to homozygotes of CC. TT homozygotes suffering from MetS had higher blood pressure values (138/86 mmHg vs. 130/77 mmHg), fasting glucose levels (6.02 vs. 5.40 mmol/L), and triglyceride levels (2.38 vs. 1.89 mmol/L) than the CC genotype. Patients with MetS and the TT genotype were characterised by higher abdominal obesity, dyslipidaemia, insulin resistance (HOMA-IR index in TT patients—3.44, CC—1.81), and HHcy (15.62 vs. 10.55 µmol/L) compared to the CC genotype [131]. 

Menopause is associated with decreased glucose tolerance, altered fat distribution, endothelial dysfunction, or abnormal plasma lipid levels. Oestrogen deficiency can also lead to reduced insulin secretion by the pancreas [132]. Therefore, in postmenopausal women, an excess of androgens may contribute to the development of insulin resistance regardless of obesity [133]. It may also be associated with elevated Hcy concentrations, as numerous studies indicate a connection between the metabolism of this amino acid and insulin resistance.

In an analysis conducted at the Menopause Clinic of the University of Athens, 84 healthy women aged 49 to 69 years were studied who had been in menopause for at least one year. The study revealed an association between the *MTHFR* polymorphism and central obesity, as well as increased androgenicity in these patients. The 677T allele (Ala222Val) was positively correlated with testosterone concentrations, FAI (free androgen index), and FEI (free oestrogen index). Furthermore, women with the 677T allele had a higher BMI (*p* = 0.027) and WHR (waist–hip ratio; *p* = 0.044) compared to women with the CC genotype [134].

The results indicating a relationship between polymorphisms in the *MTHFR* and *MTR* genes and overweight/obesity are still controversial. Researchers investigated the association of these polymorphisms in the Han population and conducted a meta-analysis involving 5431 study patients and 24,896 control patients. No significant association was found between the *MTHFR* C677T and *MTRR* A66G polymorphisms and overweight or obesity [135].

Metabolic disorders also include autosomal dominant-inherited familial hypercholesterolemia. To examine the relationship between *MTHFR* gene polymorphisms and familial hypercholesterolemia, a study was conducted involving 125 Caucasian citizens. The distribution of genotypes was as follows: CC—34%, CT—44%, and TT—22%. There was a negative correlation between Hcy levels and HDL fraction cholesterol levels. Furthermore, individuals with HHcy and the TT genotype had significantly reduced HDL fraction cholesterol levels (TT—1.14 ± 0.26 mmol/L; CT—1.33 ± 0.39 mmol/L; CC—1.39 ± 0.34 mmol/L) regardless of age, BMI, sex, mutation of the LDLR gene (low-density lipoprotein receptor), or vitamin B_12_ and folic acid levels [136]. 

Reduced NO bioavailability is associated with obesity, as well as impaired NO-dependent endothelial relaxation. Therefore, antioxidant medications, such as statins, which reduce Hcy levels in individuals with hyperlipidaemia, can potentially lead to improvements in endothelial dysfunction through NO mechanisms and increased endothelin-1 expression [137]. The impact of simvastatin treatment (drug of a statin group) on Hcy and nitrite levels (as a biomarker of NO bioavailability) and the influence of the 677C>T polymorphism on the modification of treatment effects were examined. Twenty-five women with BMI≥30 kg/m² were administered a daily dose of 20 mg of simvastatin for six weeks. Women with obesity with the T variant of the *MTHFR* gene were hypothesised to show a greater reduction in Hcy levels after simvastatin treatment, and this effect may lead to increased NO synthesis. Simvastatin treatment significantly reduced total cholesterol, LDL fraction cholesterol, and Hcy concentrations and also increased nitrite concentration (up to 60.9%). Although simvastatin reduced Hcy levels in both groups (CC and T carriers), the extent of the decrease in Hcy levels was more pronounced in individuals with the T allele (from 9.4 ± 2.9 to 7.5 ± 1.0 µmol/L) compared to patients with the CC genotype (from 7.6 ± 1.9 to 6.8 ± 1.5 µmol/L). However, the Hcy concentrations were comparable between both groups after treatment. Similarly, prior to the intervention, nitrite concentrations were markedly elevated in individuals who had the CC genotype when contrasted with those who carried the T allele. However, after treatment, these levels did not show significant differences between the groups. Obese women without comorbidities carrying the T variant of the C677T polymorphism were shown to experience better benefits from simvastatin treatment, particularly in terms of increased levels of NO [138]. 

The link between obesity and the C677T polymorphism may also be due to impaired folic acid metabolism and its reduced bioavailability in patients with the TT genotype [6]. This hypothesis is supported by a National Health and Nutrition Examination Survey (NHANES III; 1988-1994) that shows an association between low folic acid concentrations and high BMI in women of childbearing age. For every 10 kg/m^2^ increase in BMI, there was a 15.6% decrease in plasma folic acid concentration [139]. However, the NHANES 2007–2010 do not show a correlation between BMI and erythrocyte folate concentrations (indicator of body folate stores) [140]. 

The disruption in folate metabolism influenced by the presence of *MTHFR* polymorphisms is associated with an interruption in lipoprotein metabolism [141]. Therefore, researchers have begun searching for explanations of obesity in the context of impaired folate metabolism. Terruzzi et al. [141] in 2007 examined the association of multiple polymorphisms: *MTHFR* C677T and A1298C, *MTR* A2756G, *MTRR* A66G, *BHMT* G742A, and *CBS* 68-bp ins. Polymorphic variants were determined in 54 subjects with a normal BMI (22.4 ± 1.8 kg/m^2^) and in 82 obese patients (BMI 34.1 ± 7.1 kg/m^2^). Hcy, folic acid, and vitamins B_6_ and B_12_ did not show significant variance. However, leptin concentration exhibited a significant increase (*p* = 0.005) in the obese patients in contrast to the control group. A significant association was detected between genotypes AC of the *MTHFR* gene (OR = 2.5), AG of the *MTR* gene (OR = 3.0), and AG of the *MTRR* gene and the development of obesity. Furthermore, the risk remained remarkably higher for combined genotypes. However, a similar correlation was not observed for the C677T polymorphism. The researchers suggest that metabolism of Hcy is impaired in obese patients due to disrupted enzymatic activity of MTHFR, MTR, and MTRR, which are responsible for providing donors of the methyl group. This suggests that these polymorphisms serve as genetic risk factors for the development of obesity [142]. The interplay between studied polymorphisms and lipid profiles can be influenced by nutrition. Healthy diets possess the capacity to positively impact lipid profiles, thus offering effective solutions for managing dyslipidaemia and targeting biochemical pathways. This has the potential to alleviate the impact of genetic variability on lipid metabolism in individuals.

## 7. Polymorphism of *MTHFR* and Its Relationship with Hyperhomocysteinemia, Oxidative Stress, and Lipoprotein Modification

The C677T polymorphism of the *MTHFR* gene results in a 50–60% reduction in enzyme activity [143]. This polymorphism is associated with increased oxidative stress, which presents as an imbalance between pro-oxidant and antioxidant processes. This imbalance plays an important role in the pathogenesis of many diseases and is a major contributor to endothelial dysfunction [144]. To restore homeostasis and prevent endothelial damage caused by ROS accumulation, cells intensively carry out the detoxification process, which consumes significant amounts of glutathione (GSH), an important antioxidant. However, in the presence of the GSH 677TT genotype, the depletion is higher. This is because under conditions of high Hcy levels, this amino acid undergoes autooxidation, thus disrupting the trans-sulphuration pathway responsible for the crucial synthesis of GSH [144]. This results in an increase in cellular damage. With advancing age, the accumulation of toxins and the increasing damage from oxidative stress contribute to the development of a wide range of diseases and accelerate the ageing process. 

Oxidative stress marker concentrations and *MTHFR* genotype analysis were performed in a group of 66 Egyptian beta-thalassemia patients aged 8 to 26 years. Among patients with the TT genotype, a significant reduction in HDL cholesterol levels and the HDL/LDL ratio was observed compared to patients with the CC genotype (*p* < 0.05). Additionally, plasma levels of oxLDL (oxidised low-density lipoprotein) levels were found to be significantly higher in patients with TT and CT genotypes than in those with the CC genotype (*p* < 0.05). Plasma concentrations of MDA (malondialdehyde) were significantly higher in TT patients, and concentrations of NOx (total nitric oxide) and TAC (total antioxidant capacity) were significantly reduced compared to the CC genotype (*p* < 0.05). These data highlight the possible involvement of the C677T polymorphism in influencing the development of pro-oxidant diseases in individuals with TT homozygosity. This observation aligns with the results of previous research studies [145,146].

In the context of obesity, irregular lipid metabolism can cause health issues, such as hypertension, hyperlipidaemia, and diabetes. Furthermore, this disrupted lipid metabolism plays a role in the production of reactive oxygen and nitrogen species (RONS), which encompass superoxide radicals, hydrogen peroxide (H_2_O_2_), hydroxyl radicals, and nitric oxide radicals. The primary origins of intracellular reactive oxygen species (ROS) include mitochondria and NADPH oxidases, while additional contributors encompass eNOS uncoupled, cytochrome P450, xanthine oxidase (XO), the endoplasmic reticulum (ER), peroxidases, and cyclooxygenases. The interaction between these ROS sources can intensify oxidative stress, thus leading to cellular impairment and dysfunction [147]. According to the hypothesis of “kindling radicals”, the primary production of RONS triggers secondary damage, such as eNOS uncoupling. In this process, electrons “leak” from the transport chain and are transported to molecular oxygen, resulting in the formation of a peroxynitrite ion instead of nitric oxide [148]. 

Oxidative stress is closely related to cancer, CVD, and neurodegenerative disorders, including Alzheimer’s disease. H_2_O_2_ is classified as an ROS, and it is found in high concentrations in the brain. It appears that neurones have a stress sensor/regulator. PKCγ (protein kinase C isoform γ) has a zinc finger domain. The oxidation of cysteine, which enters this domain, triggers the activation of PKC. The activated kinase is then translocated to the cell membrane and phosphorylates the gap junction proteins. This process is crucial for disassembling gap junction plaques during oxidative stress. Inhibition of gap junctions in response to oxidative stress could provide a temporary “stress-protective” effect to cells by preventing the transmission of apoptotic signals to neighbouring cells through open gap junctions [149]. The presence of PKC in brain tissues appears to prevent brain ischemia and is a target of ischemic preconditioning [149]. This is especially important in post-stroke patients. Stroke, which encompasses both acute IS and intracerebral haemorrhage, leads to the demise of neuronal cells and the release of elements, such as damage-associated molecular patterns (DAMPs), which trigger localised inflammation in the affected area of the brain. Global cerebral inflammation can permanently shape the pathophysiology of post-stroke brain damage and promote a decrease in global brain functions, such as cognitive function [150].

Inflammatory cells, such as neutrophils, granulocytes, and macrophages, as well as T cells and dendritic cells that possess NADPH oxidase activity, are capable of generating RONS or at least activating phagocytic cells, thus resulting in an “oxidative burst” in the bloodstream [151]. Hcy also demonstrates pro-oxidant properties, as research has indicated that both Hcy and HCTL elevated ROS levels in human ARPE-19 cells (human retinal pigment epithelial cells). ARPE-19 cells were treated with prooxidants, including 50 μg/mL of oxLDL, 500 μM of Hcy, 500 nM of HCTL, 100 μg/mL of AGE (advanced glycation end products), and 200 μM of H_2_O_2_. The levels of pro-inflammatory cytokines (IL-6, IL-8) were measured because elevated oxidative stress can induce an inflammatory response. HCTL-treated cells were shown to exhibit the highest release of IL-6. The tested metabolites activated the NF–κB pathway, leading to pro-apoptotic changes in ARPE-19 cells [152]. Furthermore, a study conducted by Barathi et al. revealed that both Hcy and HCTL are responsible for the accumulation of MDA in vascular endothelial cells, thus serving as an indicator of oxidative stress [153]. Hcy disrupts the metabolism by cyclising to form HCTL, which leads to homocysteinylation of proteins, including LDL. According to research, incubation of LDL with 100 μmol/L of HCTL for 2 h leads to homocysteinylation of approximately 10% of lysyl residues in apoB-100 [154]. Thiolated LDL is taken up by macrophages and HCTL is released from homocysteinylated LDL within the wall of the blood vessel. This process promotes endothelial membrane injury, oxidation of cholesterol and unsaturated lipids, platelet aggregation, deposition of sulphated glycosaminoglycans, fibrosis, and calcification of atherosclerotic plaques [155]. 

HCTL levels were measured in patients with MTHFR deficiency and CBS deficiency. Plasma HCTL levels in individuals with TT genotypes (11.8 ± 8.8 nM) of the *MTHFR* gene were significantly higher compared to heterozygous CT individuals (0.5 ± 0.29 nM) or wild-type (CC genotype) individuals (0.2 ± 0.14 nM). [156]. High concentrations of Hcy also promote the modification of LDL particles by inducing their oxidation. Elevated levels of ox-LDL are associated with an increased risk of atherosclerosis, making these modified lipoproteins indicators of this disease [64]. A study involving 115 Brazilian adolescents (10–19 years) who had an elevated risk of CVD demonstrated that the presence of the T allele in the *MTHFR* gene results in increased levels of oxLDL levels (CT + TT—9.9 ± 20.5 U/L, CC—3.9 ± 4.5 U/L). Adolescents with the T allele also showed a tendency to have a larger waist circumference compared to those with the wild-type allele. HDL concentrations were also found to be significantly lower in adolescents who had Hcy concentrations in the higher tertile compared to those in the lower tertile [157]. This is possibly related to the suppression of gene transcription for apolipoprotein A-1, the main protein component of HDL [158], and decreased HDL production in the liver [159]. Microarrays and RT-PCR analysis revealed reduced mRNA biosynthesis for ApoA-I and ApoA-IV and increased mRNA for cholesterol 7 α-hydroxylase (rate-limiting enzyme in cholesterol conversion to bile acids) in the liver of Mthfr^+/−^ mice compared with Mthfr^+/+^ mice. Mthfr^+/−^ mice exhibited a decrease in Mthfr enzyme activity of 52% (in the liver) and 62% (in plasma) of the concentration found in mice with the wild-type genotype [158]. Furthermore, a negative correlation was observed between ApoA-I and plasma Hcy concentrations, as well as between HDL cholesterol levels and plasma Hcy concentrations in men with coronary heart disease [158]. Studies of HepG2 cells treated with Hcy at 5mmol/L revealed reduced levels of peroxisome proliferator activated receptor (PPAR) α and ApoA-I protein and decreased ApoA-I promoter activity. Furthermore, the study revealed an increased expression of both phosphorylated and nonphosphorylated forms of MTHFR after transfecting HepG2 cells with PPAR. This suggests that PPAR may be involved in the regulation of ApoA-I and possibly even the MTHFR enzyme itself [158].

## 8. Epigenetic Modifications and the C677T Polymorphism of the *MTHFR* Gene

The proper functioning of enzymes in folate metabolism is essential for the synthesis of methyl group donors, which are responsible for establishing the correct DNA methylation pattern. Reduced methylation at specific DNA sites is associated with activation of the transcription process, whereas increased methylation is associated with the inhibition of transcription. Hence, polymorphic gene variants encoding Hcy/methyl cycle enzymes can influence gene expression by altering DNA methylation [160]. Therefore, the connection between inadequate folic acid intake and overweight/obesity may derive from epigenetic regulation of gene expression. Dietary habits, such as the intake of dietary folic acid, which is crucial for providing methyl groups necessary for DNA methylation processes, also have a substantial impact. It has been observed that reducing the intake of folic acid in humans leads to a decrease in genomic DNA methylation. The research comprised an analysis of leukocytes obtained from the peripheral blood of healthy, postmenopausal women. During a 7-week period, the individuals had a diet that was moderately low in folate (118 µg/d). Following this period, they underwent 7 weeks of folate repletion (two groups receiving either 200 µg or 415 µg of folate daily). Incorporation of [^3^H]methyl groups increased significantly (*p* = 0.0025) in response to folate depletion, suggesting undermethylation of DNA. Furthermore, the reduced rate of DNA methylation observed at week 7 was associated with decreased serum folate levels and significantly higher plasma total homocysteine (tHcy) concentrations compared to initial measurements [161]. This research offers proof that insufficient folate intake leads to a decrease in the methylation of leukocyte DNA and suggests that the DNA methylation status could function as a marker of folate levels.

Methylation also plays a crucial role in regulating the balance of concentrations of SAM, SAH, choline, phosphatidylcholine (PC), and phosphatidylethanolamine (PE). The methylation of PE is the primary process that consumes SAM, and when this process is impaired, it leads to an increase in the SAM/SAH ratio and disrupts the overall methylation capacity of the cell, ultimately causing an excess of histone methylation. The methylation of PE for PC synthesis in the liver is of great significance because PC is crucial for liver secretion of lipoproteins and bile [162]. 

The consumption of saturated fatty acids leads to an increased synthesis of PC from PE through the action of phosphatidylethanolamine methyltransferase (PEMT), which can result in elevated Hcy concentrations. PEMT consumes three molecules of SAM, resulting in an equal number of SAH molecules, which are subsequently converted to Hcy by SAH hydrolase. SAH has a high affinity for the catalytic centre of most SAM-dependent methyltransferases [163] and is a potential inhibitor of enzymes involved in transmethylation processes, including MTHFR. Therefore, consistent hydrolysis of SAH to Hcy and adenosine is essential to support the proper methylation of DNA, RNA, proteins, phospholipids, histones, and neurotransmitters. In addition, elevated SAH concentrations in plasma and lymphocytes have been linked to increased DNA hypomethylation in lymphocytes [164]. The functional outcomes resulting from reduced cellular methylation are considerable and encompass various effects. These include the onset of nerve demyelination in the CNS, decreased synthesis of neurotransmitters, decreased macrophage capacity for chemotaxis and phagocytosis, and changes in cell membrane fluidity. 

A study focused on investigating how the C677T polymorphism in the *MTHFR* gene directly affects DNA methylation. The researchers assessed genomic DNA methylation in a group of 19 patients; of these patients, 9 had the CC genotype, and 10 were TT homozygotes. They used an enzymatic assay that measures the capacity of DNA to accept methyl groups in vitro. DNA with less methylation in vivo has a greater ability to accept radioactively labelled methyl groups in vitro. Patients with the TT genotype showed a greater methylation capacity (12,615 ± 1836 dpm/2 microg of DNA) compared to CC homozygotes (7843 ± 1043 dpm/2 microg of DNA; *p* < 0.05). This means that DNA from individuals with the TT genotype is hypomethylated compared to DNA from individuals with the wild type. Folic acid concentration in erythrocytes was also noted to be correlated with DNA methylation capacity in patients with the TT genotype [165]. Global DNA hypomethylation is also relevant in certain conditions, such as hypertension. A study investigating the influence of the C677T polymorphism on global methylation was carried out in a cohort of 218 hypertensive patients and 263 control subjects. Only cases without medication had significantly lower global DNA methylation levels compared to controls (*p* = 0.05). In cases, regardless of treatment, there was a substantial disparity of 5 mC% among the three genotypes (CC, CT, and TT), while no such difference was observed in the control group. Specifically, cases not on medication with the TT genotype exhibited significantly reduced methylation levels compared to the TT genotype within the control group and the treated cases (*p* < 0.01). Global DNA hypomethylation induced by hypertension can result in various metabolic disadvantages, particularly in relation to cardiovascular health, due to the genome’s instability. However, pharmaceutical interventions can help alleviate or reverse this condition [166].

## 9. Conclusions and Future Perspectives

Elevated levels of homocysteine, known as hyperhomocysteinemia, have emerged as a significant risk factor for various cardiovascular and metabolic diseases. The C677T polymorphism of the MTHFR gene, associated with increased Hcy levels in the blood, may have an impact on the risk of civilisation diseases. For individuals identified as carriers of this polymorphism, adjusting diet and lifestyle factors is crucial to reduce the overall risk of pathological states. Numerous studies suggest that polymorphisms in the *MTHFR* gene, especially the C677T variant, are linked to a higher risk of developing cardiovascular disease, hypertension, diabetes, overweight, and obesity. However, this impact is not consistent and is contingent on several factors, such as ethnic background, dietary habits, or the existence of other gene variations involved in homocysteine metabolism and methylation processes. However, more research should be conducted with larger cohorts in diverse geographic regions and ethnic groups to comprehensively establish the relationship between this gene and the risk of diseases. 

## Data Availability

Not applicable.

## References

[1] (2023). National Center for Biotechnology Information (NCBI). https://www.ncbi.nlm.nih.gov/.

[2] Homberger A., Linnebank M., Winter C., Willenbring H., Marquardt T., Harms E., Koch H.G. (2000). Genomic structure and transcript variants of the human methylenertolate reductase gene. Eur. J. Hum. Genet..

[3] Goyette P., Pai A., Milos R., Frosst P., Tran P., Chen Z., Chan M., Rozen R. (1998). Gene structure of human and mouse methylenetetrahydrofolate reductase (MTHFR). Mamm. Genome.

[4] Gaughan D.J., Barbaux S., Kluijtmans L.A., Whitehead A.S. (2000). The human and mouse methylenetetrahydrofolate reductase (MTHFR) genes: Genomic organization, mRNA structure and linkage to the CLCN6 gene. Gene.

[5] Tran P., Leclerc D., Chan M., Pai A., Hiou-Tim F., Wu Q., Goyette P., Artigas C., Milos R., Rozen R. (2002). Multiple transcription start sites and alternative splicing in the methylenetetrahydrofolate reductase gene result in two enzyme isoforms. Mamm. Genome.

[6] Frosst P., Blom H.J., Milos R., Goyette P., Sheppard C.A., Matthews R.G., Boers G.J., den Heijer M., Kluijtmans L.A., van den Heuvel L.P. (1995). A candidate genetic risk factor for vascular disease: A common mutation in methylenetetrahydrofolate reductase. Nat. Genet..

[7] Swanson D.A., Liu M.L., Baker P.J., Garrett L., Stitzel M., Wu J., Harris M., Banerjee R., Shane B., Brody L.C. (2001). Targeted disruption of the methionine synthase gene in mice. Mol. Cell. Biol..

[8] Mentch S.J., Locasale J.W. (2016). One-carbon metabolism and epigenetics: Understanding the specificity. Ann. N. Y. Acad. Sci..

[9] Chiang P.K., Gordon R.K., Tal J., Zeng G.C., Doctor B.P., Pardhasaradhi K., McCann P.P. (1996). S-Adenosylmethionine and methylation. FASEB J..

[10] Selhub J. (1999). Homocysteine metabolism. Annu. Rev. Nutr..

[11] Mudd S.H., Uhlendorf B.W., Freeman J.M., Finkelstein J.D., Shih V.E. (1972). Homocystinuria associated with decreased methylenetetrahydrofolate reductase activity. Biochem. Biophys. Res. Commun..

[12] Kang S.S., Zhou J., Wong P.W., Kowalisyn J., Strokosch G. (1988). Intermediate homocysteinemia: A thermolabile variant of methylenetetrahydrofolate reductase. Am. J. Hum. Genet..

[13] Kang S.S., Wong P.W., Bock H.G., Horwitz A., Grix A. (1991). Intermediate hyperhomocysteinemia resulting from compound heterozygosity of methylenetetrahydrofolate reductase mutations. Am. J. Hum. Genet..

[14] Raza S.T., Abbas S., Ahmed F., Fatima J., Zaidi Z.H., Mahdi F. (2012). Association of MTHFR and PPARγ2 gene polymorphisms in relation to type 2 diabetes mellitus cases among north Indian population. Gene.

[15] Nefic H., Mackic-Djurovic M., Eminovic I. (2018). The Frequency of the 677C>T and 1298A>C Polymorphisms in the Methylenetetrahydrofolate Reductase (MTHFR) Gene in the Population. Med. Arch..

[16] Yadav U., Kumar P., Rai V. (2014). Global prevalence of MTHFR C677T gene polymorphism: A meta-analysis of population based studies. Indian J. Clin. Biochem..

[17] van der Put N.M., Gabreëls F., Stevens E.M., Smeitink J.A., Trijbels F.J., Eskes T.K., van den Heuvel L.P., Blom H.J. (1998). A second common mutation in the methylenetetrahydrofolate reductase gene: An additional risk factor for neural-tube defects?. Am. J. Hum. Genet..

[18] Matthews R.G., Daubner S.C. (1982). Modulation of methylenetetrahydrofolate reductase activity by S-adenosylmethionine and by dihydrofolate and its polyglutamate analogues. Adv. Enzym. Regul..

[19] Sumner J., Jencks D.A., Khani S., Matthews R.G. (1986). Photoaffinity labeling of methylenetetrahydrofolate reductase with 8-azido-S-adenosylmethionine. J. Biol. Chem..

[20] Yamada K., Chen Z., Rozen R., Matthews R.G. (2001). Effects of common polymorphisms on the properties of recombinant human methylenetetrahydrofolate reductase. Proc. Natl. Acad. Sci. USA.

[21] Froese D.S., Kopec J., Rembeza E., Bezerra G.A., Oberholzer A.E., Suormala T., Lutz S., Chalk R., Borkowska O., Baumgartner M.R. (2018). Structural basis for the regulation of human 5,10-methylenetetrahydrofolate reductase by phosphorylation and S-adenosylmethionine inhibition. Nat. Commun..

[22] Yamada K., Strahler J.R., Andrews P.C., Matthews R.G. (2005). Regulation of human methylenetetrahydrofolate reductase by phosphorylation. Proc. Natl. Acad. Sci. USA.

[23] Zheng Y., Ramsamooj S., Li Q., Johnson J.L., Yaron T.M., Sharra K., Cantley L.C. (2019). Regulation of folate and methionine metabolism by multisite phosphorylation of human methylenetetrahydrofolate reductase. Sci. Rep..

[24] Patanwala I., King M.J., Barrett D.A., Rose J., Jackson R., Hudson M., Philo M., Dainty J.R., Wright A.J., Finglas P.M. (2014). Folic acid handling by the human gut: Implications for food fortification and supplementation. Am. J. Clin. Nutr..

[25] Scaglione F., Panzavolta G. (2014). Folate, folic acid and 5-methyltetrahydrofolate are not the same thing. Xenobiotica.

[26] Liew S.C. (2016). Folic acid and diseases—Supplement it or not?. Rev. Assoc. Med. Bras..

[27] EFSA (2014). Panel on Dietetic Products, Nutrition and Allergies, Scientific Opinion on Dietary Reference Values for folate. EFSA J..

[28] Palchetti C.Z., Paniz C., de Carli E., Marchioni D.M., Colli C., Steluti J., Pfeiffer C.M., Fazili Z., Guerra-Shinohara E.M. (2017). Association between Serum Unmetabolized Folic Acid Concentrations and Folic Acid from Fortified Foods. J. Am. Coll. Nutr..

[29] Tsang B.L., Devine O.J., Cordero A.M., Marchetta C.M., Mulinare J., Mersereau P., Guo J., Qi Y.P., Berry R.J., Rosenthal J. (2015). Assessing the association between the methylenetetrahydrofolate reductase (MTHFR) 677C>T polymorphism and blood folate concentrations: A systematic review and meta-analysis of trials and observational studies. Am. J. Clin. Nutr..

[30] Nishio K., Goto Y., Kondo T., Ito S., Ishida Y., Kawai S., Naito M., Wakai K., Hamajima N. (2008). Serum folate and methylenetetrahydrofolate reductase (MTHFR) C677T polymorphism adjusted for folate intake. J. Epidemiol..

[31] Siri P.W., Verhoef P., Kok F.J. (1998). Vitamins B6, B12, and folate: Association with plasma total homocysteine and risk of coronary atherosclerosis. J. Am. Coll. Nutr..

[32] Bagley P.J., Selhub J. (1998). A common mutation in the methylenetetrahydrofolate reductase gene is associated with an accumulation of formylated tetrahydrofolates in red blood cells. Proc. Natl. Acad. Sci. USA.

[33] Hiraoka M., Kagawa Y. (2017). Genetic polymorphisms and folate status. Congenit. Anom..

[34] Lucock M. (2004). Is folic acid the ultimate functional food component for disease prevention?. Br. Med. J..

[35] Hum D.W., MacKenzie R.E. (1991). Expression of active domains of a human folate-dependent trifunctional enzyme in *Escherichia coli*. Protein Eng..

[36] Blom H.J., Smulders Y. (2011). Overview of homocysteine and folate metabolism. With special references to cardiovascular disease and neural tube defects. J. Inherit. Metab. Dis..

[37] Bailey L.B., Gregory J.F. (1999). Polymorphisms of methylenetetrahydrofolate reductase and other enzymes: Metabolic significance, risks and impact on folate requirement. J. Nutr..

[38] Wolffe A.P., Matzke M.A. (1999). Epigenetics: Regulation through repression. Science.

[39] Avendaño C., Menéndez J.C. (2008). Chapter 2—Antimetabolites. Medicinal Chemistry of Anticancer Drugs.

[40] Ankar A., Kumar A. (2022). Vitamin B12 Deficiency.

[41] Baltaci D., Kutlucan A., Turker Y., Yilmaz A., Karacam S., Deler H., Ucgun T., Kara J.H. (2013). Association of vitamin B12 with obesity, overweight, insulin resistance and metabolic syndrome, and body fat composition; primary care-based study. Med. Glas..

[42] Baltaci D., Kutlucan A., Öztürk Ş., Karabulut I., Yildirim H., Celer A., Celbek G., Kara J. (2012). Evaluation of vitamin B12 level in middle-aged obese women with metabolic and nonmetabolic syndrome: Case-control study. Turk. J. Med. Sci..

[43] Alemán G., Tovar A.R., Torres N. (2001). [Homocysteine metabolism and risk of cardiovascular diseases: Importance of the nutritional status on folic acid, vitamins B6 and B12]. Rev. Investig. Clin..

[44] Malinow M.R. (1990). Hyperhomocyst(e)inemia. A common and easily reversible risk factor for occlusive atherosclerosis. Circulation.

[45] Baszczuk A., Kopczyński Z. (2014). Hyperhomocysteinemia in patients with cardiovascular disease. Adv. Hyg. Exp. Med..

[46] Tavakkoly Bazzaz J., Shojapoor M., Nazem H., Amiri P., Fakhrzadeh H., Heshmat R., Parvizi M., Hasani Ranjbar S., Amoli M.M. (2010). Methylenetetrahydrofolate reductase gene polymorphism in diabetes and obesity. Mol. Biol. Rep..

[47] Upchurch G.R., Welch G.N., Fabian A.J., Freedman J.E., Johnson J.L., Keaney J.F., Loscalzo J. (1997). Homocyst(e)ine decreases bioavailable nitric oxide by a mechanism involving glutathione peroxidase. J. Biol. Chem..

[48] Gellekink H., den Heijer M., Heil S.G., Blom H.J. (2005). Genetic determinants of plasma total homocysteine. Semin. Vasc. Med..

[49] Chen P., Poddar R., Tipa E.V., Dibello P.M., Moravec C.D., Robinson K., Green R., Kruger W.D., Garrow T.A., Jacobsen D.W. (1999). Homocysteine metabolism in cardiovascular cells and tissues: Implications for hyperhomocysteinemia and cardiovascular disease. Adv. Enzyme Regul..

[50] Khajuria A., Houston D.S. (2000). Induction of monocyte tissue factor expression by homocysteine: A possible mechanism for thrombosis. Blood.

[51] Kaplan P., Tatarkova Z., Sivonova M.K., Racay P., Lehotsky J. (2020). Homocysteine and Mitochondria in Cardiovascular and Cerebrovascular Systems. Int. J. Mol. Sci..

[52] Yamamoto M., Hara H., Adachi T. (2000). Effects of homocysteine on the binding of extracellular-superoxide dismutase to the endothelial cell surface. FEBS Lett..

[53] Chang L., Geng B., Yu F., Zhao J., Jiang H., Du J., Tang C. (2008). Hydrogen sulfide inhibits myocardial injury induced by homocysteine in rats. Amino Acids.

[54] Longoni A., Kolling J., Siebert C., Dos Santos J.P., da Silva J.S., Pettenuzzo L.F., Meira-Martins L.A., Gonçalves C.A., de Assis A.M., Wyse A.T. (2017). 1,25-Dihydroxyvitamin D(3) prevents deleterious effects of homocysteine on mitochondrial function and redox status in heart slices. Nutr. Res..

[55] Wu X., Zhang L., Miao Y., Yang J., Wang X., Wang C.C., Feng J., Wang L. (2019). Homocysteine causes vascular endothelial dysfunction by disrupting endoplasmic reticulum redox homeostasis. Redox Biol..

[56] Navneet S., Cui X., Zhao J., Wang J., Kaidery N.A., Thomas B., Bollinger K.E., Yoon Y., Smith S.B. (2019). Excess homocysteine upregulates the NRF2-antioxidant pathway in retinal Müller glial cells. Exp. Eye Res..

[57] Sibrian-Vazquez M., Escobedo J.O., Lim S., Samoei G.K., Strongin R.M. (2010). Homocystamides promote free-radical and oxidative damage to proteins. Proc. Natl. Acad. Sci. USA.

[58] Lang D., Kredan M.B., Moat S.J., Hussain S.A., Powell C.A., Bellamy M.F., Powers H.J., Lewis M.J. (2000). Homocysteine-induced inhibition of endothelium-dependent relaxation in rabbit aorta: Role for superoxide anions. Arterioscler. Thromb. Vasc. Biol..

[59] Mudd S.H., Finkelstein J.D., Refsum H., Ueland P.M., Malinow M.R., Lentz S.R., Jacobsen D.W., Brattström L., Wilcken B., Wilcken D.E. (2000). Homocysteine and its disulfide derivatives: A suggested consensus terminology. Arterioscler. Thromb. Vasc. Biol..

[60] Castro R., Rivera I., Blom H.J., Jakobs C., Tavares de Almeida I. (2006). Homocysteine metabolism, hyperhomocysteinaemia and vascular disease: An overview. J. Inherit. Metab. Dis..

[61] Sacco R.L., Adams R., Albers G., Alberts M.J., Benavente O., Furie K., Goldstein L.B., Gorelick P., Halperin J., Harbaugh R. (2006). Guidelines for prevention of stroke in patients with ischemic stroke or transient ischemic attack: A statement for healthcare professionals from the American Heart Association/American Stroke Association Council on Stroke: Co-sponsored by the Council on Cardiovascular Radiology and Intervention: The American Academy of Neurology affirms the value of this guideline. Circulation.

[62] Naruszewicz M. (2008). Homocysteina jako czynnik ryzyka chorób cywilizacyjnych; w jakich przypadkach konieczne jest jej oznaczanie?. Chor. Serca I Naczyń.

[63] Seo H., Oh H., Park H., Park M., Jang Y., Lee M. (2010). Contribution of dietary intakes of antioxidants to homocysteine-induced low density lipoprotein (LDL) oxidation in atherosclerotic patients. Yonsei Med. J..

[64] Lentz S.R., Sadler J.E. (1991). Inhibition of thrombomodulin surface expression and protein C activation by the thrombogenic agent homocysteine. J. Clin. Investig..

[65] Nishinaga M., Shimada K. (1994). Heparan sulfate proteoglycan of endothelial cells: Homocysteine suppresses anticoagulant active heparan sulfate in cultured endothelial cells. Rinsho Byori.

[66] Hajjar K.A. (1993). Homocysteine-induced modulation of tissue plasminogen activator binding to its endothelial cell membrane receptor. J. Clin. Investig..

[67] Fan X., Zhang L., Li H., Chen G., Qi G., Ma X., Jin Y. (2020). Role of homocysteine in the development and progression of Parkinson’s disease. Ann. Clin. Transl. Neurol..

[68] Buysschaert M., Dramais A.S., Wallemacq P.E., Hermans M.P. (2000). Hyperhomocysteinemia in type 2 diabetes: Relationship to macroangiopathy, nephropathy, and insulin resistance. Diabetes Care.

[69] Joshi M., Baipadithaya G., Balakrishnan A., Hegde M., Vohra M., Ahamed R., Nagri S., Ramachandra L., Satyamoorthy K. (2016). Elevated homocysteine levels in type 2 diabetes induce constitutive neutrophil extracellular traps. Sci. Rep..

[70] Yilmaz N. (2012). Relationship between paraoxonase and homocysteine: Crossroads of oxidative diseases. Arch. ed. Sci..

[71] Jakubowski H., Goldman E. (1993). Synthesis of homocysteine thiolactone by methionYL-TRNA synthetase in cultured mammalian cells. FEBS Lett..

[72] Silla Y., Varshney S., Ray A., Basak T., Zinellu A., Sabareesh V., Carru C., Sengupta S. (2019). Hydrolysis of homocysteine thiolactone results in the formation of Protein-Cys-S-S-homocysteinylation. Proteins.

[73] Exner M., Hermann M., Hofbauer R., Hartmann B., Kapiotis S., Gmeiner B. (2002). Homocysteine promotes the LDL oxidase activity of ceruloplasmin. FEBS Lett..

[74] Sengupta S., Wehbe C., Majors A.K., Ketterer M.E., DiBello P.M., Jacobsenet D.W. (2001). Relative roles of albumin and ceruloplasmin in the formation of homocystine, homocysteine-cysteine-mixed disulfide, and cystine in circulation. J. Biol. Chem..

[75] Capasso R., Sambri I., Cimmino A., Salemme S., Lombardi C., Acanfora F., Satta E., Puppione D.L., Perna A.F., Ingrosso D. (2012). Homocysteinylated albumin promotes increased monocyte-endothelial cell adhesion and up-regulation of MCP1, Hsp60 and ADAM17. PLoS ONE.

[76] Perła-Kaján J., Twardowski T., Jakubowski H. (2007). Mechanisms of homocysteine toxicity in humans. Amino Acids.

[77] Palmer R.M., Ferrige A.G., Moncada S. (1987). Nitric oxide release accounts for the biological activity of endothelium-derived relaxing factor. Nature.

[78] Moncada S. (1999). Nitric oxide: Discovery and impact on clinical medicine. J. R. Soc. Med..

[79] Yang Y.M., Huang A., Kaley G., Sun D. (2009). eNOS uncoupling and endothelial dysfunction in aged vessels. Am. J. Physiol. Heart Circ. Physiol..

[80] Arlouskaya Y., Sawicka A., Głowala M., Giebułtowicz J., Korytowska N., Tałałaj M., Nowicka G., Wrzosek M. (2019). Asymmetric Dimethylarginine (ADMA) and Symmetric Dimethylarginine (SDMA) Concentrations in Patients with Obesity and the Risk of Obstructive Sleep Apnea (OSA). J. Clin. Med..

[81] Matté C., Mackedanz V., Stefanello F.M., Scherer E.B., Andreazza A.C., Zanotto C., Moro A.M., Garcia S.C., Gonçalves C.A., Erdtmann B. (2009). Chronic hyperhomocysteinemia alters antioxidant defenses and increases DNA damage in brain and blood of rats: Protective effect of folic acid. Neurochem. Int..

[82] Li Q., Lancaster J.R. (2012). A Conspectus of Cellular Mechanisms of Nitrosothiol Formation from Nitric Oxide. For. Immunopathol. Dis. Ther..

[83] Stamler J.S., Osborne J.A., Jaraki O., Rabbani L.E., Mullins M., Singel D., Loscalzo J. (1993). Adverse vascular effects of homocysteine are modulated by endothelium-derived relaxing factor and related oxides of nitrogen. J. Clin. Investig..

[84] Homocysteine Studies Collaboration (2002). Homocysteine and risk of ischemic heart disease and stroke: A meta-analysis. J. Am. Med. Assoc..

[85] Jacques P.F., Bostom A.G., Williams R.R., Ellison R.C., Eckfeldt J.H., Rosenberg L.H., Selhub J., Rozen R. (1996). Relation between folate status, a common mutation in methylenetetrahydrofolate reductase, and plasma homocysteine concentrations. Circulation.

[86] Wan L., Li Y., Zhang Z., Sun Z., He Y., Li R. (2018). Methylenetetrahydrofolate reductase and psychiatric diseases. Transl. Psychiatry.

[87] Lloyd-Jones D., Adams R.J., Brown T.M., Carnethon M., Dai S., De Simone G., Ferguson T.B., Ford E., Furie K., Gillespie C. (2010). Heart disease and stroke statistics—2010 update: A report from the American Heart Association. Circulation.

[88] Xuan C., Bai X.Y., Gao G., Yang Q., He G.W. (2011). Association between polymorphism of methylenetetrahydrofolate reductase (MTHFR) C677T and risk of myocardial infarction: A meta-analysis for 8,140 cases and 10,522 controls. Arch. Med. Res..

[89] Li L., Yang Y., Wu S., Deng X., Li J., Ning N., Hou X. (2016). Meta-analysis of association between MTHFR C677T polymorphism and risk of myocardial infarction: Evidence from forty-four case-control studies. Int. J. Clin. Exp. Med..

[90] Dalen J.E., Alpert J.S., Goldberg R.J., Weinstein R.S. (2014). The epidemic of the 20(th) century: Coronary heart disease. Am. J. Med..

[91] Shan J.G., Xue S. (2016). MTHFR C677T polymorphism and coronary artery disease risk in the Chinese population: A meta-analysis based on 33 studies. Int. J. Clin. Exp. Med..

[92] Hao L., Ma J., Stampfer M.J., Ren A., Tian Y., Tang Y., Willett W.C., Li Z. (2003). Geographical, seasonal and gender differences in folate status among Chinese adults. J. Nutr..

[93] Klerk M., Verhoef P., Clarke R., Blom H.J., Kok F.J., Schouten F.G. (2002). MTHFR 677C-->T polymorphism and risk of coronary heart disease: A meta-analysis. J. Am. Med. Assoc..

[94] Fan S., Yang B., Zhi X., Wang Y., Wei J., Zheng Q., Sun G. (2016). Interactions of Methylenetetrahydrofolate Reductase C677T Polymorphism with Environmental Factors on Hypertension Susceptibility. Int. J. Environ. Res. Public. Health.

[95] Hou J., Zeng X., Xie Y., Wu H., Zhao P. (2018). Genetic polymorphisms of methylenetetrahydrofolate reductase C677T and risk of ischemic stroke in a southern Chinese Hakka population. Medicine.

[96] Liu L., Wang D., Wong K.S., Wang Y. (2011). Stroke and stroke care in China: Huge burden, significant workload, and a national priority. Stroke.

[97] Goracy I., Cyrylowski L., Kaczmarczyk M., Fabian A., Koziarska D., Goracy J., Ciechanowicz A. (2009). C677T polymorphism of the methylenetetrahydrofolate reductase gene and the risk of ischemic stroke in Polish subjects. J. Appl. Genet..

[98] Lv Q.Q., Lu J., Sun H., Zhang J.S. (2015). Association of methylenetetrahydrofolate reductase (MTHFR) gene polymorphism with ischemic stroke in the E astern Chinese Han population. Genet. Mol. Res..

[99] Alves-Silva J.M., Zuzarte M., Girão H., Salgueiro L. (2021). The Role of Essential Oils and Their Main Compounds in the Management of Cardiovascular Disease Risk Factors. Molecules.

[100] Lima L.M., Carvalho M., Fernandes A.P., Sabino Ade P., Loures-Vale A.A., da Fonseca Neto C.P., Garcia J.C., Saad J.A., Sousa M.O. (2007). Homocysteine and methylenetetrahydrofolate reductase in subjects undergoing coronary angiography. Arq. Bras. Cardiol..

[101] Hales C.M., Carroll M.D., Fryar C.D., Ogden C.L. (2020). Prevalence of Obesity and Severe Obesity Among Adults: United States, 2017–2018.

[102] Tremblay A., Pérusse L., Bouchard C. (2004). Energy balance and body-weight stability: Impact of gene-environment interactions. Br. J. Nutr..

[103] Lewis S.J., Lawlor D.A., Nordestgaard B.G., Tybjaerg-Hansen A., Ebrahim S., Zacho J., Ness A., Leary S., Smith G.D. (2008). The methylenetetrahydrofolate reductase C677T genotype and the risk of obesity in three large population-based cohorts. Eur. J. Endocrinol..

[104] Wrzosek M., Ślusarczyk K. (2022). Methylenetetrahydrofolate Reductase C677T Gene Variant in Relation to Body Mass Index and Folate Concentration in a Polish Population. Biomedicines.

[105] Leal-Ugarte E., Peralta V., Meza-Espinoza J.P., Duran J., Macias-Gomez N., Bocanegra-Alonso A., Lara-Ramos J. (2019). Association of the MTHFR 677C>T Polymorphism with Obesity and Biochemical Variables in a Young Population of Mexico. J. Med. Biochem..

[106] Pirozzi F.F., Belini Junior E., Okumura J.V., Salvarani M., Bonini-Domingos C.R., Ruiz M.A. (2018). The relationship between of ACE I/D and the MTHFR C677T polymorphisms in the pathophysiology of type 2 diabetes mellitus in a population of Brazilian obese patients. Arch. Endocrinol. Metab..

[107] Bastard J.P., Maachi M., Lagathu C., Kim M.J., Caron M., Vidal H., Capeau J., Feve B. (2006). Recent advances in the relationship between obesity, inflammation, and insulin resistance. Eur. Cytokine Netw..

[108] Tilg H., Moschen A.R. (2006). Adipocytokines: Mediators linking adipose tissue, inflammation and immunity. Nat. Rev. Immunol..

[109] Caputo T., Gilardi F., Desvergne B. (2017). From chronic overnutrition to metaflammation and insulin resistance: Adipose tissue and liver contributions. FEBS Lett..

[110] Livshits G., Kato B.S., Wilson S.G., Spector T.D. (2007). Linkage of genes to total lean body mass in normal women. J. Clin. Endocrinol. Metab..

[111] Di Renzo L., Rizzo M., Iacopino L., Sarlo F., Domino E., Jacoangeli F., Colica C., Sergi D., De Lorenzo A. (2013). Body composition phenotype: Italian Mediterranean Diet and C677T MTHFR gene polymorphism interaction. Eur. Rev. Med. Pharmacol. Sci..

[112] Frelut M.L., Nicolas J.P., Guilland J.C., de Courcy G.P. (2011). Methylenetetrahydrofolate reductase 677 C->T polymorphism: A link between birth weight and insulin resistance in obese adolescents. Int. J. Pediatr. Obes..

[113] Ong K.K. (2006). Size at birth, postnatal growth and risk of obesity. Horm. Res..

[114] Powell-Wiley T.M., Poirier P., Burke L.E., Després J.P., Gordon-Larsen P., Lavie C.J., Lear S.A., Ndumele C.E., Neeland I.J., Sanders P. (2021). Obesity and Cardiovascular Disease: A Scientific Statement from the American Heart Association. Circulation.

[115] Huang T., Yuan G., Zhang Z., Zou Z., Li D. (2008). Cardiovascular pathogenesis in hyperhomocysteinemia. Asia Pac. J. Clin. Nutr..

[116] Berstad P., Konstantinova S.V., Refsum H., Nurk E., Vollset S.E., Tell G.S., Ueland P.M., Drevon C.A., Ursin G. (2007). Dietary fat and plasma total homocysteine concentrations in 2 adult age groups: The Hordaland Homocysteine Study. Am. J. Clin. Nutr..

[117] Kucukhuseyin O., Kurnaz O., Akadam-Teker A.B., Isbir T., Bugra Z., Ozturk O., Yilmaz-Aydogan H. (2013). The association of MTHFR C677T gene variants and lipid profiles or body mass index in patients with diabetic and nondiabetic coronary heart disease. J. Clin. Lab. Anal..

[118] Barnes A.S. (2011). The epidemic of obesity and diabetes: Trends and treatments. Tex. Heart Inst. J..

[119] Li Y., Zhang H., Jiang C., Xu M., Pang Y., Feng J., Xiang X., Kong W., Xu G., Li Y. (2013). Hyperhomocysteinemia promotes insulin resistance by inducing endoplasmic reticulum stress in adipose tissue. J. Biol. Chem..

[120] Ozcan U., Cao Q., Yilmaz E., Lee A.H., Iwakoshi N.N., Ozdelen E., Tuncman G., Görgün C., Glimcher L.H., Hotamisligil G.S. (2004). Endoplasmic reticulum stress links obesity, insulin action, and type 2 diabetes. Science.

[121] Li Y., Jiang C., Xu G., Wang N., Zhu Y., Tang C., Wang X. (2008). Homocysteine upregulates resistin production from adipocytes in vivo and in vitro. Diabetes.

[122] Fernández-Sánchez A., Madrigal-Santillán E., Bautista M., Esquivel-Soto J., Morales-González A., Esquivel-Chirino C., Durante-Montiel I., Sánchez-Rivera G., Valadez-Vega C., Morales-González J.A. (2011). Inflammation, oxidative stress, and obesity. Int. J. Mol. Sci..

[123] Lunegova O., Kerimkulova A., Turdakmatov N., Sovkhozova N., Nabiev M., Isakova Z., Iusupova É., Moldokeeva C., Gotfrid I., Mirrakhimov A. (2011). Association of C677T Gene Polymorphism of Methylenetetrahydrofolate Reductase with Insulin Resistance Among Kirghizes. Kardiologiia.

[124] Schettini M.A.S., Passos R.F.D.N., Koike B.D.V. (2023). Shift Work and Metabolic Syndrome Updates: A Systematic Review. Sleep. Sci..

[125] Kheradmand M., Maghbooli Z., Salemi S., Sanjari M. (2017). Associations of MTHFR C677T polymorphism with insulin resistance, results of NURSE Study (Nursing Unacquainted Related Stress Etiologies). J. Diabetes Metab. Disord..

[126] Zhi X., Yang B., Fan S., Li Y., He M., Wang D., Wang Y., Wei J., Zheng Q., Sun G. (2016). Additive interaction of MTHFR C677T and MTRR A66G polymorphisms with being overweight/obesity on the risk of type 2 diabetes. Int. J. Environ. Res. Public Health.

[127] Dobrowolski P., Prejbisz A., Kuryłowicz A., Baska A., Burchardt P., Chlebus K., Dzida G., Jankowski P., Jaroszewicz J., Jaworski P. (2022). Metabolic syndrome—A new definition and management guidelines: A joint position paper by the Polish Society of Hypertension, Polish Society for the Treatment of Obesity, Polish Lipid Association, Polish Association for Study of Liver, Polish Society of Family Medicine, Polish Society of Lifestyle Medicine, Division of Prevention and Epidemiology Polish Cardiac Society, “Club 30” Polish Cardiac Society, and Division of Metabolic and Bariatric Surgery Society of Polish Surgeons. Arch. Med. Sci..

[128] Canale M.P., Manca di Villahermosa S., Martino G., Rovella V., Noce A., De Lorenzo A., Di Daniele N. (2013). Obesity-related metabolic syndrome: Mechanisms of sympathetic overactivity. Int. J. Endocrinol..

[129] Chen A.R., Zhang H.G., Wang Z.P., Fu S.J., Yang P.Q., Ren J.G., Ning Y.Y., Hu X.J., Tian L.H. (2010). C-reactive protein, vitamin B12 and C677T polymorphism of N-5,10-methylenetetrahydrofolate reductase gene are related to insulin resistance and risk factors for metabolic syndrome in Chinese population. Clin. Investig. Med..

[130] Ellingrod V.L., Miller D.D., Taylor S.F., Moline J., Holman T., Kerr J. (2008). Metabolic syndrome and insulin resistance in schizophrenia patients receiving antipsychotics genotyped for the methylenetetrahydrofolate reductase (MTHFR) 677C/T and 1298A/C variants. Schizophr. Res..

[131] Wang J., Xu L., Xia H., Li Y., Tang S. (2018). Association of MTHFR C677T gene polymorphism with metabolic syndrome in a Chinese population: A case-control study. J. Int. Med. Res..

[132] Rosano G.M., Vitale C., Marazzi G., Volterrani M. (2007). Menopause and cardiovascular disease: The evidence. Climacteric.

[133] Lee C.C., Kasa-Vubu J.Z., Supiano M.A. (2004). Androgenicity and obesity are independently associated with insulin sensitivity in postmenopausal women. Metabolism.

[134] Lambrinoudaki I., Kaparos G., Papadimitriou D., Sergentanis T.N., Creatsa M., Alexandrou A., Logothetis E., Christodoulakos G., Kouskouni E. (2008). Methylenetetrahydrofolate reductase C677T polymorphism is associated with central adiposity and increased androgenicity in healthy postmenopausal women. Eur. J. Endocrinol..

[135] Fan S.J., Yang B.Y., Zhi X.Y., He M., Wang D., Wang Y.X., Wang Y.N., Wei J., Zheng Q.M., Sun G.F. (2015). Are MTHFR C677T and MTRR A66G Polymorphisms Associated with Overweight/Obesity Risk? From a Case-Control to a Meta-Analysis of 30,327 Subjects. Int. J. Mol. Sci..

[136] Real J.T., Martinez-Hervas S., Garcia-Garcia A.B., Chaves F.J., Civera M., Ascaso J.F., Carmena R. (2009). Association of C677T polymorphism in MTHFR gene, high homocysteine and low HDL cholesterol plasma values in heterozygous familial hypercholesterolemia. J. Atheroscler. Thromb..

[137] Jiang S., Chen Q., Venners S.A., Zhong G., Hsu Y.H., Xing H., Wang X., Xu X. (2013). Effect of simvastatin on plasma homocysteine levels and its modification by MTHFR C677T polymorphism in Chinese patients with primary hyperlipidemia. Cardiovasc. Ther..

[138] Villela M.P., Andrade V.L., Eccard B., Jordao A.A., Sertorio J.T., Tanus-Santos J.E., Silva L.F., Silveira J.N., Sandrim V.C. (2014). Homocysteine and nitrite levels are modulated by MTHFR 677C>T polymorphism in obese women treated with simvastatin. Clin. Exp. Pharmacol. Physiol..

[139] Mojtabai R. (2004). Body mass index and serum folate in childbearing age women. Eur. J. Epidemiol..

[140] Manandhar M., Beydoun H., Kancherla V. (2020). Association between body mass index and folate insufficiency indicative of neural tube defects risk among nonpregnant women of childbearing age in the United States, NHANES, 2007–2010. Birth Defects Res..

[141] Semmler A., Moskau S., Grigull A., Farmand S., Klockgether T., Smulders Y., Blom H., Zur B., Stoffel-Wagner B., Linnebank M. (2010). Plasma folate levels are associated with the lipoprotein profile: A retrospective database analysis. Nutr. J..

[142] Terruzzi I., Senesi P., Fermo I., Lattuada G., Luzi L. (2007). Are genetic variants of the methyl group metabolism enzymes risk factors predisposing to obesity?. J. Endocrinol. Invest..

[143] Raghubeer S., Matsha T.E. (2021). Methylenetetrahydrofolate (MTHFR), the One-Carbon Cycle, and Cardiovascular Risks. Nutrients.

[144] de Oliveira R.P.D., da Silva E.G., de Faria Santos K., da Silva Santos R., da Silva Reis A.A. (2023). The combined effects of GSTM1/GSTT1 and MTHFR C677T polymorphisms on the systemic arterial hypertension susceptibility: A genetic association study in Brazilian diabetic patients. Hum. Gene.

[145] Abd-Elmawla M.A., Rizk S.M., Youssry I., Shaheen A.A. (2016). Impact of Genetic Polymorphism of methylenetetrahydrofolate reductase C677T on Development of Hyperhomocysteinemia and Related Oxidative Changes in Egyptian Beta-Thalassemia Major PatiBeta. PLoS ONE.

[146] Pitsavos C., Panagiotakos D., Trichopoulou A., Chrysohoou C., Dedoussis G., Chloptsios Y., Choumerianou D., Stefanadis C. (2006). Interaction between Mediterranean diet and methylenetetrahydrofolate reductase C677T mutation on oxidized low density lipoprotein concentrations: The ATTICA study. Nutr. Metab. Cardiovasc. Dis..

[147] de Almeida A., de Oliveira J., da Silva Pontes L.V., de Souza Júnior J.F., Gonçalves T.A.F., Dantas S.H., de Almeida Feitosa M.S., Silva A.O., de Medeiros L.A. (2022). ROS: Basic Concepts, Sources, Cellular Signaling, and its Implications in Aging Pathways. Oxid. Med. Cell Longev..

[148] Karbach S., Wenzel P., Waisman A., Munzel T., Daiber A. (2014). eNOS uncoupling in cardiovascular diseases--the role of oxidative stress and inflammation. Curr. Pharm. Des..

[149] Lin D., Takemoto D.J. (2005). Oxidative activation of protein kinase Cgamma through the C1 domain. Effects on gap junctions. J. Biol. Chem..

[150] Shi K., Tian D.C., Li Z.G., Ducruet A.F., Lawton M.T., Shi F.D. (2019). Global brain inflammation in stroke. Lancet Neurol..

[151] Cave A.C., Brewer A.C., Narayanapanicker A., Ray R., Grieve D.J., Walker S., Shah A.M. (2006). NADPH oxidases in cardiovascular health and disease. Antioxid. Redox Signal.

[152] AnandBabu K., Sen P., Angayarkanni N. (2019). Oxidized LDL, homocysteine, homocysteine thiolactone and advanced glycation end products act as pro-oxidant metabolites inducing cytokine release, macrophage infiltration and pro-angiogenic effect in ARPE-19 cells. PLoS ONE.

[153] Barathi S., Angayarkanni N., Pasupathi A., Natarajan S.K., Pukraj R., Dhupper M., Velpandian T., Muralidharan C., Sivashanmugham M. (2010). Homocysteinethiolactone and paraoxonase: Novel markers of diabetic retinopathy. Diabetes Care.

[154] Ferretti G., Bacchetti T., Moroni C., Vignini A., Nanetti L., Curatola G. (2004). Effect of homocysteinylation of low density lipoproteins on lipid peroxidation of human endothelial cells. J. Cell Biochem..

[155] McCully K.S. (1993). Chemical pathology of homocysteine. I. Atherogenesis. Ann. Clin. Lab. Sci..

[156] Chwatko G., Boers G.H., Strauss K.A., Shih D.M., Jakubowski H. (2007). Mutations in methylenetetrahydrofolate reductase or cystathionine beta-synthase gene, or a high-methionine diet, increase homocysteine thiolactone levels in humans and mice. FASEB J..

[157] Morais C.C., Alves M.C., Augusto E.M., Abdalla D.S., Horst M.A., Cominetti C. (2015). The MTHFR C677T Polymorphism Is Related to Plasma Concentration of Oxidized Low-Density Lipoprotein in Adolescents with Cardiovascular Risk Factors. J. Nutrigenet. Nutrigenom..

[158] Mikael L.G., Genest J., Rozen R. (2006). Elevated homocysteine reduces apolipoprotein A-I expression in hyperhomocysteinemic mice and in males with coronary artery disease. Circ. Res..

[159] Liao D., Yang X., Wang H. (2007). Hyperhomocysteinemia and high-density lipoprotein metabolism in cardiovascular disease. Clin. Chem. Lab. Med..

[160] Jones P.A., Takai D. (2001). The role of DNA methylation in mammalian epigenetics. Science.

[161] Rampersaud G., Kauwell G., Hutson A., Cerda J., Bailey L. (2000). Genomic DNA methylation decreases in response to moderate folate depletion in elderly women. Am. J. Clin. Nutr..

[162] Ye C., Sutter B.M., Wang Y., Kuang Z., Tu B.P. (2017). A Metabolic Function for Phospholipid and Histone Methylation. Mol. Cell.

[163] Hoffman D.R., Marion D.W., Cornatzer W.E., Duerre J.A. (1980). S-Adenosylmethionine and S-adenosylhomocystein metabolism in isolated rat liver. Effects of L-methionine, L-homocystein, and adenosine. J. Biol. Chem..

[164] Yi P., Melnyk S., Pogribna M., Pogribny I.P., Hine R.J., James S.J. (2000). Increase in plasma homocysteine associated with parallel increases in plasma S-adenosylhomocysteine and lymphocyte DNA hypomethylation. J. Biol. Chem..

[165] Stern L.L., Mason J.B., Selhub J., Choi S.W. (2000). Genomic DNA hypomethylation, a characteristic of most cancers, is present in peripheral leukocytes of individuals who are homozygous for the C677T polymorphism in the methylenetetrahydrofolate reductase gene. Cancer Epidemiol. Biomark. Prev..

[166] Yadav S., Longkumer I., Joshi S., Saraswathy K.N. (2021). Methylenetetrahydrofolate reductase gene polymorphism, global DNA methylation and blood pressure: A population based study from North India. BMC Med. Genom..

